# Dermal glucocorticoids are uncoupled from stress physiology and infection

**DOI:** 10.1093/conphys/coaf005

**Published:** 2025-02-11

**Authors:** Victor Quadros, Brady Inman, Nina McDonnell, Kaitlyn Williams, L Michael Romero, Douglas C Woodhams

**Affiliations:** Department of Biology, University of Massachusetts Boston, 100 Morrissey Blvd., Boston, MA 02125, USA; Department of Biology, University of Massachusetts Boston, 100 Morrissey Blvd., Boston, MA 02125, USA; Department of Biology, University of Massachusetts Boston, 100 Morrissey Blvd., Boston, MA 02125, USA; Department of Biology, University of Massachusetts Boston, 100 Morrissey Blvd., Boston, MA 02125, USA; Department of Biology, Tufts University, Robinson Hall, Rm 369200 College Ave., Medford, MA 02155, USA; Department of Biology, University of Massachusetts Boston, 100 Morrissey Blvd., Boston, MA 02125, USA

**Keywords:** Amphibian, *Batrachochytrium dendrobatidis*conservation physiology, corticosterone, *Notophthalmus viridescens*, Rana (lithobates) pipiens

## Abstract

Ongoing amphibian population declines are caused by factors such as climate change, habitat destruction, pollution and infectious diseases not limited to chytridiomycosis. Unfortunately, action is taken against these factors once population collapses are underway. To avoid these *post hoc* responses, wildlife endocrinology aims to analyse physiological mediators that predict future population declines to inform wildlife management. Mediators typically investigated are stress hormones known as glucocorticoids, which are produced by the Hypothalamus—Pituitary—Interrenal axis (HPI axis). The HPI axis is the part of the endocrine system that helps amphibians cope with stress. Chronic increases in glucocorticoids due to stress can lead to immune dysfunction, which makes amphibians more susceptible to infectious diseases. Despite this predictive potential of glucocorticoids, interpretation of glucocorticoid data is confounded by sampling design and type. Glucocorticoid monitoring classically involves blood sampling, which is not widely applicable in amphibians as some are too small or delicate to sample, and repeated samples are often valued. To address this, we tried to validate skin swabbing via corticosterone (CORT) and adrenocorticotropin hormone (ACTH) injections in adults of two amphibian species: Eastern red-spotted newts, *Notophthalmus viridescens viridescens,* with natural skin infections with *Batrachochytrium dendrobatidis* (*Bd*) upon collection in the field, and Northern leopard frogs, *Rana (Lithobates) pipiens,* raised in captivity and naïve to *Bd* exposure. Further, we determined the predictive potential of skin glucocorticoids on *Bd* load in the field via correlations in Eastern red-spotted newts. We found that hormones present in the skin are not related to the HPI axis and poorly predict infection load; however, skin hormone levels strongly predicted survival in captivity. Although skin swabbing is not a valid method to monitor HPI axis function in these species, the hormones present in the skin still play important roles in organismal physiology under stressful conditions relevant to wildlife managers.

## Graphical Abstract

**Graphical Abstract:**
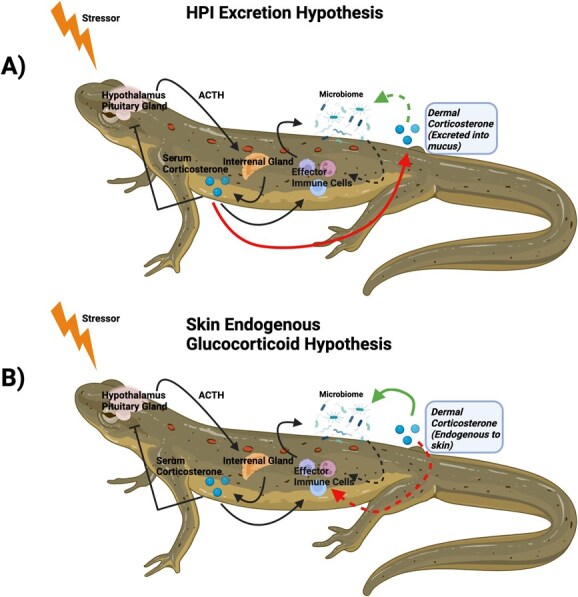

## Introduction

Along with declining global biodiversity trends, amphibian extinction rates continue to climb in the 21st century, resulting in an ongoing biodiversity crisis (Cowie *et al.*, 2022; Luedtke *et al.*, 2023; McKee *et al.*, 2004). Although all groups of vertebrates are under pressure, amphibians are particularly susceptible to the climactic and anthropogenic disturbances that are driving the biodiversity crisis, such as habitat degradation, pollution, atmospheric warming and infectious diseases (Luedtke *et al.*, 2023). As infectious agents continue to spread amongst the herpetofauna (Bosch *et al.*, 2006; Fisher & Garner, 2020), conservation physiologists need to take both responsive and proactive actions in order to protect amphibian biodiversity (Assis *et al.*, 2023; McEwen & Wingfield, 2003; Narayan *et al.*, 2019).

Proactive actions rely on predicting population collapses before they occur and understanding causal connections between biological indicators and future declines, with multi-sector readiness (Wikelski & Cooke, 2006). One class of biological indicators that have known physiological connections to organismal health are compounds known as glucocorticoids (Romero, 2004). Glucocorticoids are steroid hormones produced by the Hypothalamus–Pituitary–Adrenal/Interrenal axis (HPA/I) (Botía *et al.*, 2023; Ledón-Rettig *et al.*, 2009; Romero, 2004; Wingfield & Romero, 2011). The HPA/I axis is classically associated with the vertebrate ‘stress response’ to social, trophic, pathogenic or environmental stressors (Wingfield & Romero, 2011). Amphibians are no exception, since all vertebrate taxa release corticosterone (CORT) or cortisol into the serum upon exposure to noxious stimuli or when injected with adrenocorticotropin hormone (ACTH): the hormone that causes the production of glucocorticoids by the interrenal glands (Leboulenger *et al.*, 1986; Selye & Collip, 1936). Glucocorticoids have many downstream effects, such as altering immune system function, increasing blood glucose via shifts in metabolism and changes in behaviour or reproductive activity (Cannon, 1915; Selye & Collip, 1936). As a consequence, there are cases where glucocorticoids were used to predict population declines in certain taxa, such as the impact of an oil spill on marine iguanas, *Amblyrhynchus cristatus* (Wikelski *et al.*, 2001). Despite this predictive potential, the effects these hormones have depended on the amounts produced, their frequency, the species or population being investigated and the timing of hormone release (Bryant *et al.*, 2022; Chambers, 2011; Denver, 2021; Jackson *et al.*, 2021; Kirschman *et al.*, 2018; van Kesteren *et al.*, 2019).

In addition to physiological cofactors that can influence the effect glucocorticoids have on an organism; hormone sampling methods are incredibly diverse and can further impact the measurements. The classic method for measuring and monitoring glucocorticoid levels involves obtaining serum samples from multiple individuals at varying time points following capture restraint (Romero & Wingfield, 2015). Although used across a wide range of taxa, this method is only applicable to organisms that are large enough to obtain serum samples without euthanasia and organisms that can be handled safely (Narayan, 2013; Narayan *et al.*, 2019). To circumvent this, researchers have been able to obtain glucocorticoid samples from faeces (Hadinger *et al.*, 2015), hair (Sheriff *et al.*, 2011), nails (Fokidis *et al.*, 2023), feathers (Freeman & Newman, 2018; Gormally *et al.*, 2020) whale spout and baleen (Thompson *et al.*, 2014; Trumble *et al.*, 2018), aquarium water (McClelland & Woodley, 2021; Tornabene *et al.*, 2021a, 2021b), urine (Narayan & Hero, 2011; Sadoul & Geffroy, 2019) and saliva (Garde & Hansen, 2005; Hammond *et al.*, 2018). This broad range of sample types falls under the category of non-invasive methods (Narayan *et al.*, 2019; Romero & Wingfield, 2015). Non-invasive methods are important for monitoring endangered or at-risk species of amphibians, as many species that are susceptible to infectious diseases are too small to draw serum samples from (Narayan, 2013; Narayan *et al.*, 2019). Despite the potential convenience for researchers and amphibians alike, the physiological interpretation of non-invasively sampled glucocorticoids is a challenge, as the biologically active compound is present only in the serum (Romero, 2004; Smith & Vale, 2006). As more non-invasive methods arise, rigorous physiological validation of each method is required to better understand the causal connection between non-invasively sampled glucocorticoids and future population losses.

Prior investigation has showcased the potential to obtain glucocorticoid samples via dorsoventral swabbing of amphibian skin and mucus; however, interpretation of the data is limited by the use of too few biological replicates (Santymire *et al.*, 2018). Later work attempted to further validate this technique, though with mixed results (Santymire *et al.*, 2021; Scheun *et al.*, 2019), perhaps indicating species-specific physiology. Dermal swabbing aims to monitor HPI function; however, clinical research in humans has demonstrated that epidermal cells synthesize their own Pro-opiomelanocortin (POMC), ACTH and Cortisol (Hannen *et al.*, 2017; Jozic *et al.*, 2014; Phan *et al.*, 2021; Vukelic *et al.*, 2011). If this is occurring in amphibians, then dermal swabbing would not be appropriate for monitoring HPI function, or at least have substantial amounts of signal masked by the skin’s endocrine activity.

Glucocorticoids are known to affect disease processes in complex ways in various taxa, but the relationship between HPI function and chytridiomycosis outcome is under ongoing investigation; though research is limited by hormone sampling techniques available for each taxon (Fonner *et al.*, 2017; Gabor *et al.*, 2015, 2018; Pereira *et al.*, 2023; Peterson *et al.*, 2013; Searle *et al.*, 2014). Many amphibians are susceptible to chytridiomycosis, which is caused by *Batrachochytrium dendrobatidis* (*Bd*) and *Batrachochytrium salamandrivorans* (*Bsal*; (Fisher & Garner, 2020). A variety of factors can influence species or population specific outcomes, such as immune repertoire (Assis *et al.*, 2023; Grogan *et al.*, 2018; Murone *et al.*, 2016; Pereira *et al.*, 2023), skin microbiome composition (Colombo *et al.*, 2015; Knutie *et al.*, 2018, 2018; Woodhams *et al.*, 2014), pollutant exposure (Bosch *et al.*, 2021; Buck *et al.*, 2015; Jiménez *et al.*, 2021), climate change (Bosch *et al.*, 2006; Bradley *et al.*, 2019) and timing of infection (Bosch *et al.*, 2021; Kirschman *et al.*, 2018).

Since monitoring *Bd* and *Bsal* involves obtaining dermal swabs to obtain DNA to quantify through quantitative PCR (qPCR) (Boyle *et al.*, 2004), monitoring skin glucocorticoids concomitantly would be simple to implement in the field. Before dermal swabbing can be implemented in the field, we need to determine if skin glucocorticoids picked up by swabs are related to HPI function. Even if dermal glucocorticoids are unrelated to HPI function, if they still can predict survival or infection load, then the swabbing technique could still have a role in predicting outcomes in amphibians threatened by disease. Therefore, the questions addressed by this study are threefold:

1) Do skin swabs adequately uptake glucocorticoids?
2) Are dermal glucocorticoids related to HPI axis activity? Do they respond to acute stress?
3) Can dermal glucocorticoids predict infection load or survival?

To test whether skin glucocorticoids are related to HPI function, Eastern red-spotted newts, *Notophthalmus viridescens viridescens* (hereafter, Eastern newts), and Northern leopard frogs, *Rana* (*Lithobates*) *pipiens* (hereafter, Northern leopard frogs), were selected as models due to their small size, high abundance, and sensitivity to disease and anthropogenic disturbances (Bouchard *et al.*, 2009). Adults of both species were sampled. The Eastern newts had natural skin infections with *Bd* upon collection in the field, and the frogs were raised in outdoor mesocosms from frog eggs, thus naïve to *Bd* exposure. The swabs used on both amphibians were evaluated for potential matrix effects by spiking solutions with known amounts of CORT and swabbing them. CORT was the primary hormone studied due to the lack of detection of cortisol in the skin of both amphibian species even during the injection challenge. Groups of Eastern newts and Northern leopard frogs were injected with saline solution, ACTH and CORT, and then swabbed repeatedly over a 24-h period to determine if CORT in the skin is coupled to HPI function. Finally, *Bd* load and survival were monitored in Eastern newts and statistically tested for correlation with dermal CORT.

## Materials and Methods

### Swab matrix validation, hormone extraction and enzyme-linked immunosorbent assays

**Figure 1:**
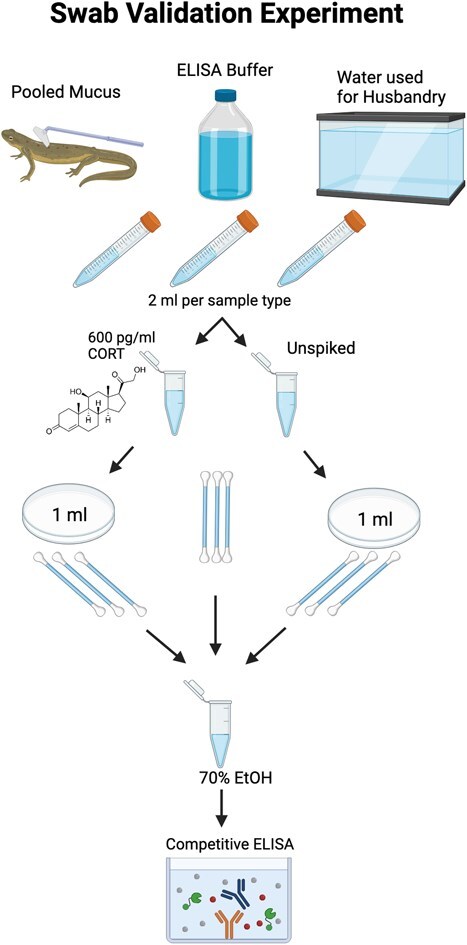
Visualization of the swab matrix validation protocol utilized in the current study. This figure was made using Biorender.

To determine if rayon swabs can effectively adsorb steroid hormones, we conducted a study using three different types of swabbing medium samples. This included mucus collected from 12 captive Eastern newts, water with the same parameters as the newts’ housing and enzyme-linked immunosorbent assay (ELISA) buffer (Cayman Chemical Company). ELISA buffer served as a positive control, as the spiked CORT is known to be chemically stable whilst dissolved in the ELISA buffer. Housing water and mucus were chosen as experimental groups because both medium types would end up on the swabs when swabbing any amphibian. Newt mucus was collected in a 50-ml conical tube using a cell scraper to gently rub the mucus off the newt skin. To do this, we restrained newts by their tails with their heads facing towards the inside of the tube. After holding the conical at an angle to allow the newts to rest their stomachs at the top, we gently scraped their dorsal and ventral surfaces. Once mucus coated the scraper, the newt was removed and placed back into its respective holding tub and the mucus was smeared onto the bottom of the conical. Since we house aquatic Eastern newts, some housing water got into the sample, although this reflects what would be obtained through dermal swabbing if an adult newt were immediately swabbed after capture from an aquatic habitat in the field. ELISA buffer (1X) was made from a 10X stock diluted into MiliQ water according to instructions from the CORT Cayman Chemical ELISA kit. Water used to house Eastern newts was obtained from a reservoir tank in the animal care facility containing reverse osmosis water reconstituted with dilute salts and sodium hydroxide to a pH range of 7.4–7.6 and conductivity of 300 μs/cm. In total, 800 μl of each medium type was collected (Fig. 1).

We also included three swabs that were not used to swab anything (‘neat’) as a control to measure the background produced by the swab itself. Each swab type (excluding ‘neat’ swabs) was further split into two 400-μl samples: one spiked with 600 pg/ml of CORT, and one without any spike. We placed 400 μl of each sample type into sterile Petri dishes and used three sterile rayon CLASSICQSwabs to rub the surface of the dish. The medium was streaked across the dish surface three times before flipping the swabs over and swabbing it three more times. Next, we placed the swabs into 1.5-ml microcentrifuge tubes containing 500 μl of 70% ethanol and stored them at 4°C overnight for hormone extraction (Fig. 1).

The next day, we removed the swabs from the ethanol solution and split the ‘neat’ swab samples into two 250-μl samples: one spiked with 2500 pg/ml of CORT into the ethanol and one without a spike to determine extraction efficiency. We then placed all tubes into a SpeedVac and allowed the ethanol to evaporate under vacuum for 3 h at 60°C. We reconstituted the samples in 200 μl of 1X ELISA buffer (Cayman Chemical Company), vortexed them for 1 min, sonicated them for 20 min and left them to shake at 150 rpm on an orbital shaker (Carolina Orbital Shaker, Item # 216230) for 30 min to reconstitute the hormone. Finally, we stored the samples at 4°C for future use.

Once all samples were reconstituted, we ran each sample on a CORT ELISA from Cayman Chemical Company. We loaded 50 μl of each sample into each well and ran them in triplicate. We read the plates on a POLARstar Omega plate reader at 412 nm according to the manufacturer’s instructions. We recorded the resulting absorbance values on a spreadsheet. All samples were run ‘neat’.

### Field swabs, sampling and husbandry

To measure the natural variation in CORT obtained through dermal swabbing, we collected 35 adult Eastern red-spotted newts, *N. viridescens viridescens*, from the George D. Aiken Wilderness in Woodford, Vermont, in May 2023. The site consisted of a high-altitude, low-pH sphagnum bog that is situated next to a trail with little tree cover. Adult Eastern newts were captured by dip-netting and each handled with a new pair of latex gloves and promptly swabbed for baseline CORT. We recorded their snout-vent length (SVL) (mean = 40.5 mm, standard deviation = 0.3 mm), weights (mean = 1.98 g, standard deviation = 0.29 g) and sex (most were in breeding condition), then placed them into sterile Whirlpack bags containing 30 ml of sterile artificial pond water for the collection of dermal mucus, which was used for a separate project. After 30 min, we re-swabbed the newts for CORT and swabbed for both *Bd* and the microbiome. We then placed them into Whirlpack bags containing water from the surrounding bog and transported them back to the lab in a cooler. After 6 h, we swabbed the newts for CORT and drip-acclimated them onto a flow-through aquarium (Aquaneering) system, housing each newt individually. The *Bd*/microbiome swabs were obtained using sterile Dryswabs from medicalwire (MW 113) and CORT swabs were obtained using CLASSIQSwabs from Copan Diagnostics.

On the Aquaneering system, we housed the Eastern newts at 18°C and changed the water twice a week. The newts were kept in 0.8-l holding tanks with 3 × 3 cm styrofoam floats and PVC tube hides. After each water change, we fed them bloodworms thawed in system water and allowed them to acclimate for 3 weeks before conducting experiments. Dermal swabs were obtained each week to gauge changes in dermal CORT over time whilst individual survival was monitored for 2.5 months after capture. After initial mortality plateaued during acclimation, only non-diseased newts were used in experiments.

Northern leopard frog, *R.* (*Lithobates) pipiens*, eggs were collected from Shelburne Bay, Shelburne, Vermont, in April of 2023 for a separate project that was occurring concomitantly. We reared the eggs in a 15°C Conviron environmental chamber (Controlled Environments, Pembina, North Dakota) with a 12-h light cycle to replicate natural developmental conditions. Whilst the eggs were developing, we prepared outdoor mesocosms by filling 1136-l tanks with 950 l of local water and 22.5 kg of natural play sand (Pavestone, Atlanta Georgia), and covering them with 40% shade cloth. After 2 weeks, we introduced 500 ml of concentrated zooplankton from a local pond to each tank. Once tadpoles reached the free-swimming stage, each mesocosm was stocked with 20 tadpoles, for a density of 0.021 individuals per litre. Mesocosms were supplemented with 6.5 g of Aquatic Gel Diet Herbivorous Fish Formula (Mazuri Exotic Animal Nutrition, St. Louis, Missouri) every other week. As tadpoles metamorphosed throughout the spring and summer, the resulting froglets were transferred to a separate 1136-l mesocosm. This tank was held at an angle to create aquatic and terrestrial habitats with a planted margin to allow the frogs to hide. We fed frogs calcium-dusted crickets three times per week, *ad libitum*. Once all frogs reached an SVL of 1 cm, they were used for experimentation, with 27 Northern leopard frogs that were not used in any experimental manipulations being selected for the current study. All frogs were naïve to *Bd* exposure, which was confirmed with qPCR of both the zooplankton concentrate and swabs from individual frogs (see DNA extraction and qPCR section below).

**Figure 2:**
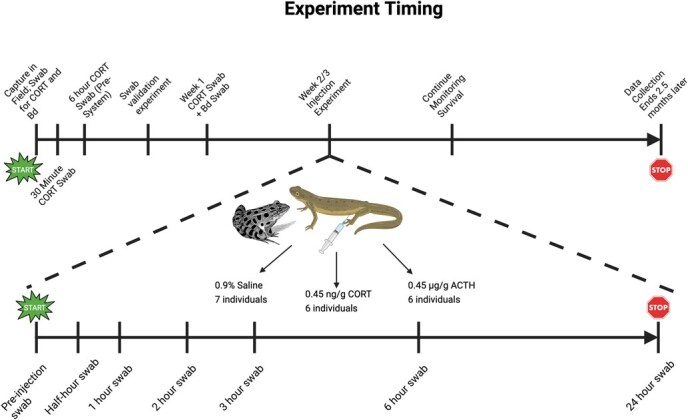
The experimental timeline of experiments with Eastern newts, *N. viridescens viridescens*, or with Northern leopard frogs, *R. (Lithobates) pipiens,* carried out over the summer of 2023. This figure was made using Biorender.

The physiological validation experiments occurred in June 2023 for newts (Fig. 2), and in October 2023 for frogs upon completion of metamorphosis and growth to minimum size.

### Newt wash

To determine if the mucus washes performed in the field had an impact on dermal CORT readings, 12 Eastern newts that had been living on the Aquaneering system for more than a year were selected for testing. One group was placed in Whirlpack bags filled with sterile artificial pond water (Wyngaard & Chinnappa, 1982), whilst the other group was returned to the holding tank they were taken from after their baseline CORT swab was obtained. After 30 min, the newts were swabbed again and put back in their holding tanks. Once the experiment was finished, the newts were given water changes and fed. All swabs were extracted as mentioned in the previous section, spiked to examine extraction efficiency, and immediately analysed using an ELISA.

### Physiological validation

Before experimentation, all newts and frogs received a 100% water change to eliminate any waterborne or faecal glucocorticoids that may have been present. The experiments began at 1000 h and finished at 1000 h the following day. The newts and frogs were not fed on the day of the experiments. Each amphibian was randomly assigned to one of three treatment groups. Roughly equal numbers of male and female Eastern newts were assigned to each treatment group; however, the sex of the Northern leopard frogs was unknown as they were too small to be reliably sexed. In the newt experiment, samples size for the control group (*n* = 7), and experimental groups CORT (*n* = 6) and ACTH injected (*n* = 6; Fig. 2) were robust for detecting significant differences in skin CORT expected based on other studies (e.g. Scheun *et al.* 2019). During the frog experiment, the ACTH group had seven frogs, whilst the other two groups had six frogs. Newts were randomly assigned to Aquaneering containers, whilst frogs were randomly assigned to different mesocosms arranged in a Latin-squares matrix, with nine bins in total. Although these sample sizes were small, we expected to see differences in CORT on the scale of 2–10 orders of magnitude between the control group and both experimental groups (Hammond *et al.*, 2018; Scheun *et al.*, 2019; Wingfield & Romero, 2011).

The first group was injected with 100 μl of 1 IU/kg synthetic ACTH (Prospec Bio HOR-279) in 0.9% saline vehicle; the second group received an injection of 100 μl of 0.446 ug/g CORT (501 320, Cayman Chemical Company) in a 0.9% saline vehicle, whilst the third group received only 0.9% saline of equal volume (Fig. 2). These concentrations were chosen according to previous work performed on both Eastern newts and Northern leopard frogs (Hammond *et al.*, 2018; Santymire *et al.*, 2018; Scheun *et al.*, 2019). Before the injections, each amphibian was swabbed with both a fine-tipped rayon swab for microbiome and Bd sample collection along with a rayon swab for a baseline measure of glucocorticoids. The corresponding glucocorticoid swabs were collected at Hours 0.5, 1, 2, 3, 4, 6 and 24 after the injection (Fig. 2). The glucocorticoid swabs were placed into 500 μl of 70% ethanol inside a microcentrifuge tube and were stored overnight at 4°C for reconstitution the next day. The microbiome swabs were stored in sterile microcentrifuge tubes and stored at −20°C until DNA extractions could be performed.

Once the swabs were reconstituted following the methods described above, they were analysed for both CORT and cortisol using the Cayman Chemical CORT ELISA kit (501320) and Cayman Chemical cortisol ELISA kit (500360). Fifty microlitres of the sample was loaded into triplicate wells. The resulting DNA from the microbiome swabs was used for Bd qPCRs to monitor disease load throughout the experiment.

### DNA extraction and qPCR

DNA was extracted from *Bd*/microbiome swabs using the gMax Mini extraction Kit (IBI Scientific). Swabs were suspended in an extraction buffer containing lysozyme followed by an incubation at 37°C for an hour. Afterward, the extraction supernatants were loaded onto spin columns, washed twice and then eluted to obtain purified DNA extracts according to the manufacturer’s instructions. All extracts were stored at −20°C for long-term storage.

To determine infection load, qPCR was performed following Boyle *et al.* (2004): 5 μl of sample was used for 25 μl total reactions using the ITS forward and reverse primers in Taqman Master Mix. To detect DNA amplification, FAM was used as the fluorescent reporter. The plates were run on a C1000 Touch Thermal Cycler (BIO-RAD).

### Data analysis and curation

All absorbance data obtained via hormone ELISAs were analysed on a spreadsheet that Cayman Chemical provides for generating standard curves to determine the concentrations of analytes. Once these concentrations were calculated, all the resulting data was placed into CSV files for analysis in R. The Cq values obtained directly from the thermocycler were converted to log ITS starting copy numbers by generating a standard curve in Microsoft Excel. Additionally, all qPCR data, field metrics and compiled hormone titres were placed into a single Excel file with multiple spreadsheets (available in Supplementary Materials: Compiled_Newt_Experimental_Data.xlxs).

For the injection series, linear mixed models made through the glmmTMB package (Brooks *et al.*, 2017) were constructed using newt ID as a random effect. Variables such as treatment, SVL, weight and sex were implemented as predictors of dermal CORT/cortisol (Supplemental Materials Section 3: Statistical Models, Supplementary Tables S1 and S2). Models with the lowest Akaike information criterion (AIC) values were selected for *post hoc* statistical testing, which consisted of 95% confidence interval (CI) generation using the emmeans package (Lenth *et al.*, 2024) to examine treatment effects. For the matrix validation assay, a suite of linear models generated using the base R linear model function were compared using the AIC, with the model generating the lowest AIC value without violating assumptions selected for further statistical analysis. Sample types, along with spiked and unspiked groups, were compared using Welch Two-Sample *t*-tests to determine if there are statistical differences associated with each sample type. All model assumptions were checked using the performance package (Lüdecke *et al.*, 2021) and all plots were generated using ggplot2 functions (Wickham *et al.*, 2019). Using the dplyr package, cleaning and rearrangement of datasets was performed (Wickham *et al.*, 2019).

The relationship between dermal CORT, disease load and survival in captivity was assessed using a mix of both linear models and Cox regressions using the ggsurvfit (Sjoberg *et al.*, 2024) and gtsummary packages (Sjoberg *et al.*, 2021) for analysis. Cox regressions were plotted according to both dermal CORT and disease load using the contsurvplot package (Denz & Timmesfeld, 2023) to eliminate any statistical manipulations that would have arisen due to the discretizing of continuous predictors. Cox regression assumptions were checked using functions from the survminer package (Kassambara *et al.*, 2024). All code and data used in this study are available on github (https://github.com/VictQuad/Dermal_CORT_Code_Data).

## Results

### Rayon swabs as a matrix

**Figure 3:**
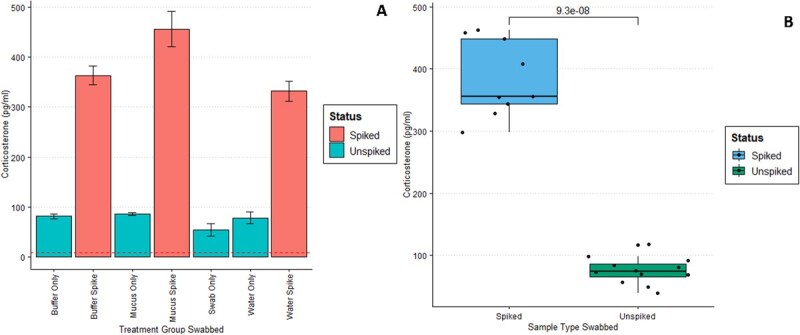
Corticosterone on rayon swabs. (a) Mean corticosterone detected according to each medium type and spike status. Vertical bars represent standard error across triplicates. All mucus was obtained from captive Eastern newts, *N. viridiescens viridescens*. The line close to the bottom of the figure indicates the lower limit of detection for the ELISA (8.8 pg/ml). (b) CORT detected according to spike status. Since all mediums were spiked with 600 pg/ml, this results in an extraction efficiency of 66.7% averaged across media. The number above the bar is the *P*-value obtained through a Welch Two-Sample *t*-test. The dots represent individual data points.

In total, 21 swabs and their corresponding extracts were collected. Across media types, spiked CORT was recovered through swabbing (Fig. 3a). Swab spike recovery was 66.7% on average, meaning that 66.7% of the 600 pg/ml spike was detected on the ELISA post-extraction across all medium types (Fig. 3b). Spiked swabs had higher detected CORT on average compared to their unspiked counterparts (*n* = 18, t = 14.426, df = 9.4638, *P* = 9.3*10^-8, Upper CI = 357.2473 pg/ml, Lower CI = 261.0176 pg/ml; Welch Two-Sample *t*-test). Extraction efficiency was 97% (Supplemental Materials: Fig. S2).

### Primary hormone, capture-restraint and washing

CORT was found in the skin of both Eastern red-spotted newts and Northern leopard frogs, whilst cortisol was not able to be detected using an ELISA (Supplemental Materials: Figs. S5 and S6). Unpublished preliminary results indicate cortisol was present below the detection limits of the ELISA based on LC–MS, and thus not present at physiologically meaningful quantities in these species (Jason Evans, *pers. comm*.).

Average dermal CORT decreased 30 min after capture in Eastern newts but returned to near-field levels after 6 h. Newts that were washed before the 30-min swab did not have any differences in dermal CORT compared to newts that were left unwashed before the 30-min swab (*n* = 11, *P* > 0.05; Supplemental Materials: Figs. S1, S3, S4).

### Dermal CORT poorly predicts infection load, yet predicts survival in captivity

**Figure 4:**
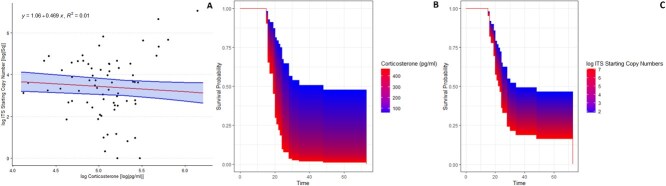
*Batrachochytrium dendrobatidis* (*Bd*) infection, dermal CORT and survival. (a) Relationship between the log of both CORT and *Bd* load indicated by ITS gene copy number. The model with the lowest AIC is fitted to the data, with the shaded region being the 95% CI around the estimated model parameters. (b) Survival curves obtained through Cox regression depicting survival probabilities associated with varying levels of dermal CORT on *N. viridescens viridescens* monitored over 2 months in captivity. (c) Survival curves obtained through the same regression as in (b), however, *Bd* infection load [log(ITS copy numbers)] was used as a predictor instead of dermal CORT.

All Eastern newts captured from the field (*n* = 35) were infected with *Bd. Bd* load in the field was 1 × 10^6^ copies on average, whilst in captivity, the average increased to 1.5 × 10^6^ copies. This 50% increase was not correlated with dermal CORT (Fig. 4a, slope = −0.255 ± 0.761 with 95% confidence, adjusted-r^2^ = 0.06; Supplemental Materials: Model Outputs 1-6). Despite this, dermal CORT on the first week in captivity strongly predicted survival after 2 months (Fig. 4b  *P* = 0.033, Score (logrank) test = 4.68, corrected regression coefficient = 1.002–1.006, standard error = 0.002, *n* = 35; Supplemental Materials: Model Outputs 1-6). *Bd* load was not as strongly predictive of survival as dermal CORT was (Fig. 4c; *P* = 0.3, Score (logrank) test = 0.03, corrected regression coefficient = 1.18–0.8478, standard error = 0.1612, *n* = 35).

### Dermal CORT is uncoupled from HPI function

**Figure 5:**
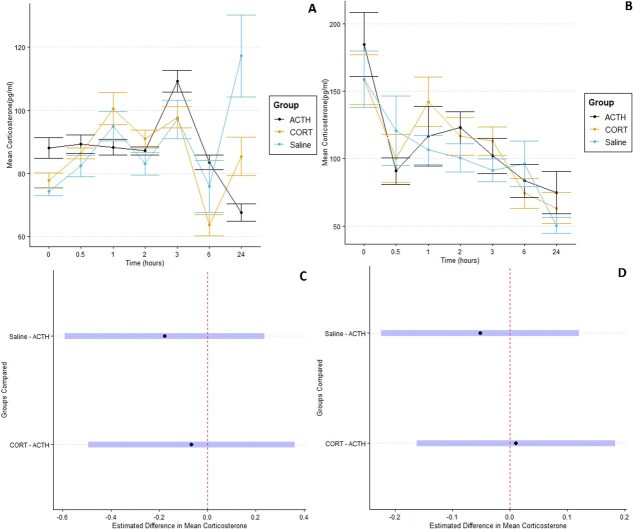
Experimental tests of HPI-axis stimulations and dermal CORT quantification. Mean estimates of Eastern newt (a) and Leopard frog (b) dermal CORT detected post-injection according to each time point sampled. Vertical bars indicate the standard error of the mean and median for each graph, respectively. Mean and median estimates were obtained from biological replicates within each injection group. (c) The 95% CIs obtained from the model with the lowest AIC examining the effects of each injection treatment on the estimated mean CORT obtained overall in the Eastern newt models. Overlap with the dotted line indicates confidence in an estimate for no contrasting effect on measured CORT when compared to another treatment group. (d) The 95% CIs obtained in the same manner as (c), except the data used to generate the above intervals was derived from Northern leopard frogs.

The model with the lowest score (ΔAIC = 0.0, DF = 11; Supplemental Materials: Table S1) was the linear mixed-effects model for Eastern newts and a simple linear model for Northern leopard frogs (ΔAIC = 0.0, DF = 9; Supplemental Materials: Table S2). During the duration of the injection series, two ACTH-injected and one CORT-injected newts perished after the third hour. Both models for newts and frogs resulted in no statistically significant differences between ACTH-, Saline- and CORT-injected amphibians (Fig. 5, *P* > 0.05 for all comparisons).

The error bars in Fig. 5a–b have substantial overlap at most time points, with the only major difference being the larger CORT detected in the saline group compared to the CORT- and ACTH-injected group at 24 h in newts. The 95% CIs estimated using treatment contrasts all overlapped with a contrast estimate of zero (Fig. 5c, d), which indicates no measured effects of injection type on dermal CORT in both Eastern newts and Northern leopard frogs.

## Discussion

### Swabs are a suitable matrix

Rayon swabs were able to recover 66.7% of the spiked CORT on average, differing from swabs that were unspiked. All medium types contributed some background on the ELISA; however, swabs on their own also contribute to this background, which can limit the sensitivity of this method at detecting dermal CORT. Despite this, if dermal CORT was responsive to HPI stimulation, then any differences in concentration >100 pg/ml would be detected. Considering that ACTH injection in amphibians can cause serum CORT or cortisol levels to increase to the nanogramme/millilitre range, we expected to detect dermal glucocorticoids within that range after a certain period if the skin mucus is linked to the HPI axis via excretion or excitation. Therefore, rayon swabs are a suitable method for collecting dermal glucocorticoids from amphibians.

### Dermal CORT and stress physiology

Our study was limited by both smaller than expected sample sizes and an inability to obtain plasma samples to directly show CORT in the serum. The smaller sample sizes came from the loss of individual animals in captivity, which ended up being a feature of the study, as survival analysis was performed, but nonetheless limited which individuals were experimented on during the physiological validation. Plasma samples would have yielded more concrete results but were impossible to obtain without lethal sampling in both species used in this study.

Despite these limitations, the CORT-injected group served as a means of avoiding lethal plasma sampling, since the measured hormone was directly placed into the serum in amounts that are beyond the natural ranges in both species (Hammond *et al.*, 2018; Pereira *et al.*, 2023). Since the bolus of CORT placed into the serum was never seen on the skin in both species, this suggests that little, if any, plasma CORT was excreted onto the skin during the injection challenge. Furthermore, ACTH injected into the serum elicited no response in dermal CORT. All of this suggests that the CORT present in the skin of both Eastern newts and Northern leopard frogs is uncoupled from CORT present in the serum. Therefore, HPI function cannot adequately be monitored using dermal swabs in these two amphibians as was previously suggested (Santymire *et al.*, 2018; Santymire *et al.*, 2021), although validated in an African amphibian, the edible bullfrog, *Pyxicephalus edulis* (Scheun *et al.*, 2019).

One caveat is that the Eastern newts were near their physiological limits due to ongoing *Bd* infections. Indeed, three newts expired during the 24-h injection experiment. In the field, most Eastern newt populations are infected with widely ranging *Bd* infection prevalence and loads (Raffel *et al.*, 2010). Thus, our results may be representative of field conditions for this species. In addition, a pilot study with healthy *Bd-*infected newts collected from the same population in summer 2022 but conducted in outdoor mesocosms also demonstrated a lack of significant impacts on dermal CORT of rough handling and filtered *Bd* metabolites when compared to control treatments (Supplemental Materials: Figs. S7–S12). Conversely, Northern leopard frogs are often not infected in the field or can recover from *Bd* infections (Wilber *et al.*, 2022). Plus, the Northern leopard frogs were raised and fully acclimatized to the environmental conditions present during experimentation, so it is highly likely that the frogs were not at their maximum hormone release rates. Despite this, there was still no signal detected in the Northern leopard frogs, suggesting uncoupling.

If dermal CORT is unrelated to the HPI axis, what explains the signal obtained from the mucus? Over the last two decades, work in humans has shown that the skin acts as its own endocrine organ, producing a wide variety of steroidal compounds or their precursors endogenously (Hannen *et al.*, 2017; Jozic *et al.*, 2014; Phan *et al.*, 2021; Vukelic *et al.*, 2011). Some of these compounds, namely POMC, ACTH and CORT, are synthesized by keratinocytes, which function independently of the HPA axis (Phan *et al.*, 2021). The uncoupling of dermal CORT from CORT present in the serum as we have shown in two amphibians here provides preliminary evidence that epidermal cells may endogenously produce CORT. To further evaluate this idea, transcriptomic data paired with immunohistochemical validation could demonstrate the endogenous production of HPI elements in the epidermis or granular glands of amphibians. In addition to immunohistochemistry, amphibian epidermal cell cultures could be used to determine if the skin is responsible for dermal glucocorticoid signals observed via skin swabbing.

### CORT, Bd infection load and survival

Dermal CORT was poorly predictive of *Bd* infection load (Fig. 4a). *Bd* infection load was weakly predictive of survival (Fig. 4b). Infection load often correlates with survival in amphibians susceptible to *Bd* chytridiomycosis (Grogan *et al.*, 2018). Since all the Eastern newts used in the experiment were infected, there were no uninfected and infected comparisons to be made, which could explain the lack of a statistical association (Fig. 4b, c; Supplemental Materials Section 3: Statistical Models) between survival and infection load. The rapid increase in infection load when newts were brought into captivity may have been the result of a release from the environmental pH constraint on *Bd* growth (Piotrowski *et al.*, 2004, Woodhams *et al.*, 2018) upon moving newts from an acidic bog to a slightly alkaline captive environment. This perhaps precipitated chytridiomycosis-associated mortality.

The relationship between dermal CORT and survival was not related to HPI stimulation. Assuming the relationship is not spurious, it could also hint at a novel role that dermal CORT plays in the physiology of amphibian skin. The Eastern newts were collected from a bog with a low pH and low conductivity but were housed in water with a pH of ~7.4 and higher conductivity. Given the shift in water parameters, it could be possible that dermal CORT participates in some form of osmoregulatory mechanism (Chambers, 2011; Smith & Vale, 2006). Recent work has demonstrated that skin glucocorticoids also influence the microbes that live on the skin in *Boana faber* (Neely *et al.*, 2023). Therefore, it is likely that microbiome assemblage shifted in concert with, and potentially in response to, dermal CORT, which can influence survival against Bd (Woodhams *et al.*, 2018). Further investigation is required to understand the physiological mechanisms underlying the observed survival data.

### Conservation implications

The urgency of developing methods to monitor and assess at-risk amphibian populations will only increase as the climate changes and as anthropogenic activity extends further into wetland habitats. Although initially promising as a non-invasive method, dermal swabs cannot adequately monitor HPI activity in two North American amphibian species. Instead, the signal obtained from dermal swabbing appears to be a consequence of the endogenous production of glucocorticoids rather than hormones excreted from the serum into the mucus. Therefore, conservationists should exercise caution when applying the dermal swabbing technique to field populations, as the technique likely monitors CORT that is not related to acute HPI-axis function. Further, this data calls attention to a need for more rigorous testing of methods as imprecise methods could lead to incorrect conclusions about wild populations. The data presented serve as both a cautionary tale and a source of further investigation, as the function of glucocorticoids in the skin across vertebrates remains a mystery.

## Supplementary Material

Web_Material_coaf005

## Data Availability

Additional information regarding statistics and prior experiments can be found in Supplemental Materials. In addition, all of the Eastern newt data are provided in a Supplementary Materials file: Compiled_Newt_Experimental_Data.xlxs, and all analyses used during the experiment along with all of the collected data are available on github (https://github.com/VictQuad/Dermal_CORT_Code_Data).

## References

[ref1] Assis VR, Robert J, Titon SCM (2023) Introduction to the special issue amphibian immunity: stress, disease and ecoimmunology. Philos Trans R Soc B: Biol Sci 378: 20220117. 10.1098/rstb.2022.0117. PMC10258669 37305915

[ref2] Bosch J, Carrascal LM, Durán L, Walker S, Fisher MC (2006) Climate change and outbreaks of amphibian chytridiomycosis in a montane area of Central Spain; is there a link? Proc R Soc B: Biol Sci 274: 253–260. 10.1098/rspb.2006.3713. PMC1685858 17148254

[ref3] Bosch J, Thumsová B, López-Rojo N, Pérez J, Alonso A, Fisher MC, Boyero L (2021) Microplastics increase susceptibility of amphibian larvae to the chytrid fungus Batrachochytrium dendrobatidis. Sci Rep 11: 22438. 10.1038/s41598-021-01973-1. 34789869 PMC8599647

[ref4] Botía M, Escribano D, Martínez-Subiela S, Tvarijonaviciute A, Tecles F, López-Arjona M, Cerón JJ (2023) Different types of glucocorticoids to evaluate stress and welfare in animals and humans: general concepts and examples of combined use. Metabolites 13. 10.3390/metabo13010106. PMC9865266 36677031

[ref5] Bouchard J, Ford A, Eigenbrod F, Fahrig L (2009) Behavioral responses of northern leopard frogs (*Rana pipiens*) to roads and traffic: implications for population persistence. Ecol Soc 14: 23. [online] URL: 10.5751/ES-03022-140223.

[ref6] Boyle DG, Boyle DB, Olsen V, Morgan JAT, Hyatt AD (2004) Rapid quantitative detection of chytridiomycosis (Batrachochytrium dendrobatidis) in amphibian samples using real-time Taqman PCR assay. Dis Aquat Organ 60: 141–148. 10.3354/dao060141. 15460858

[ref7] Bradley PW, Brawner MD, Raffel TR, Rohr JR, Olson DH, Blaustein AR (2019) Shifts in temperature influence how Batrachochytrium dendrobatidis infects amphibian larvae. PloS One 14: e0222237. 10.1371/journal.pone.0222237. 31536533 PMC6752834

[ref8] Brooks ME, Kristensen K, van Benthem KJ, Magnusson A, Berg CW, Nielsen A, Skaug HJ, Mächler M, Bolker BM (2017) glmmTMB balances speed and flexibility among packages for zero-inflated generalized linear mixed modeling. The R Journal 9: 378–400. 10.32614/RJ-2017-066.

[ref9] Bryant AR, Gabor CR, Swartz LK, Wagner R, Cochrane MM, Lowe WH (2022) Differences in corticosterone release rates of larval spring salamanders (*Gyrinophilus porphyriticus*) in response to native fish presence. Biology 11: Article 4. 10.3390/biology11040484. PMC9030379 35453684

[ref10] Buck JC, Hua J, Iii WRB, Dang TD, Urbina J, Bendis RJ, Stoler AB, Blaustein AR, Relyea RA (2015) Effects of pesticide mixtures on host-pathogen dynamics of the amphibian Chytrid fungus. PloS One 10: e0132832. 10.1371/journal.pone.0132832. 26181492 PMC4504700

[ref11] Cannon WB (1915) Bodily Changes in Pain, Hunger, Fear and Rage: An Account of Recent Researches into the Function of Emotional Excitement. New York and London D. Appleton and Company, pp. 196–200. https://dn790008.ca.archive.org/0/items/cu31924022542470/cu31924022542470.pdf.

[ref12] Chambers DL (2011) Increased conductivity affects corticosterone levels and prey consumption in larval amphibians. J Herpetol 45: 219–223. 10.1670/09-211.1.

[ref13] Colombo BM, Scalvenzi T, Benlamara S, Pollet N (2015) Microbiota and mucosal immunity in amphibians. Front Immunol 6: 111. 10.3389/fimmu.2015.00111. 25821449 PMC4358222

[ref14] Cowie RH, Bouchet P, Fontaine B (2022) The sixth mass extinction: fact, fiction or speculation? Biol Rev 97: 640–663. 10.1111/brv.12816. 35014169 PMC9786292

[ref15] Denver RJ (2021) Stress hormones mediate developmental plasticity in vertebrates with complex life cycles. Neurobiology of Stress 14: 100301. 10.1016/j.ynstr.2021.100301. 33614863 PMC7879041

[ref16] Denz R, Timmesfeld N (2023) Visualizing the (causal) effect of a continuous variable on a time-to-event outcome. Epidemiology 34: 652–660. 10.1097/EDE.0000000000001630. 37462467 PMC10392888

[ref17] Fisher MC, Garner TWJ (2020) Chytrid fungi and global amphibian declines. Nat Rev Microbiol 18: 332–343. 10.1038/s41579-020-0335-x. 32099078

[ref18] Fokidis HB, Brock T, Newman C, Macdonald DW, Buesching CD (2023) Assessing chronic stress in wild mammals using claw-derived cortisol: a validation using European badgers (Meles meles). Conserv Physiol 11: coad024. 10.1093/conphys/coad024. 37179707 PMC10171820

[ref19] Fonner CW, Patel SA, Boord SM, Venesky MD, Woodley SK (2017) Effects of corticosterone on infection and disease in salamanders exposed to the amphibian fungal pathogen Batrachochytrium dendrobatidis. Dis Aquat Organ 123: 159–171. 10.3354/dao03089. 28262636

[ref20] Freeman NE, Newman AEM (2018) Quantifying corticosterone in feathers: validations for an emerging technique. Conserv Physiol 6: coy051. 10.1093/conphys/coy051. 30323931 PMC6181252

[ref21] Gabor CR, Fisher MC, Bosch J (2015) Elevated corticosterone levels and changes in amphibian behavior are associated with Batrachochytrium dendrobatidis (Bd) infection and Bd lineage. PloS One 10: e0122685. 10.1371/journal.pone.0122685. 25893675 PMC4404099

[ref22] Gabor CR, Knutie SA, Roznik EA, Rohr JR (2018) Are the adverse effects of stressors on amphibians mediated by their effects on stress hormones? Oecologia 186: 393–404. 10.1007/s00442-017-4020-3. 29222721

[ref23] Garde AH, Hansen ÅM (2005) Long-term stability of salivary cortisol. Scand J Clin Lab Invest 65: 433–436. 10.1080/00365510510025773. 16081365

[ref24] Gormally BMG, van Rees CB, Bowers E, Reed JM, Romero LM (2020) Feather corticosterone does not correlate with environmental stressors or body condition in an endangered waterbird. Conserv Physiol 8: coaa125. 10.1093/conphys/coaa125. 33425358 PMC7772616

[ref25] Grogan LF, Robert J, Berger L, Skerratt LF, Scheele BC, Castley JG, Newell DA, McCallum HI (2018) Review of the amphibian immune response to Chytridiomycosis, and future directions. Front Immunol 9: 2018. 10.3389/fimmu.2018.02536. 30473694 PMC6237969

[ref26] Hadinger U, Haymerle A, Knauer F, Schwarzenberger F, Walzer C (2015) Faecal cortisol metabolites to assess stress in wildlife: evaluation of a field method in free-ranging chamois. Methods Ecol Evol 6: 1349–1357. 10.1111/2041-210X.12422.

[ref27] Hammond TT, Au ZA, Hartman AC, Richards-Zawacki CL (2018) Assay validation and interspecific comparison of salivary glucocorticoids in three amphibian species. Conserv Physiol 6: coy055. 10.1093/conphys/coy055. 30279992 PMC6158758

[ref28] Hannen R, Udeh-Momoh C, Upton J, Wright M, Michael A, Gulati A, Rajpopat S, Clayton N, Halsall D, Burrin J et al. (2017) Dysfunctional skin-derived glucocorticoid synthesis is a pathogenic mechanism of psoriasis. J Invest Dermatol 137: 1630–1637. 10.1016/j.jid.2017.02.984. 28359725

[ref29] Jackson MC, Pawar S, Woodward G (2021) The temporal dynamics of multiple stressor effects: from individuals to ecosystems. Trends Ecol Evol 36: 402–410. 10.1016/j.tree.2021.01.005. 33583600

[ref30] Jiménez RR, Alvarado G, Ruepert C, Ballestero E, Sommer S (2021) The fungicide chlorothalonil changes the amphibian skin microbiome: a potential factor disrupting a host disease-protective trait. Appl Microbiol 1: 26–37. 10.3390/applmicrobiol1010004.

[ref31] Jozic I, Stojadinovic O, Kirsner RS, Tomic-Canic M (2014) Stressing the steroids in skin: paradox or fine-tuning? J Invest Dermatol 134: 2869–2872. 10.1038/jid.2014.363. 25381768

[ref32] Kassambara A, Kosinski M, Biecek P, Fabian S (2024) *Survminer: drawing survival curves using “ggplot2”* (version 0.5.0) [computer software]. https://cran.r-project.org/web/packages/survminer/index.html.

[ref33] van Kesteren F, Delehanty B, Westrick SE, Palme R, Boonstra R, Lane JE, Boutin S, McAdam AG, Dantzer B (2019) Experimental increases in glucocorticoids alter function of the HPA axis in wild red squirrels without negatively impacting survival and reproduction. Physiol Biochem Zool 92: 445–458. 10.1086/705121. 31365306

[ref34] Kirschman LJ, Crespi EJ, Warne RW (2018) Critical disease windows shaped by stress exposure alter allocation trade-offs between development and immunity. J Anim Ecol 87: 235–246. 10.1111/1365-2656.12778. 29095486

[ref35] Knutie SA, Gabor CR, Kohl KD, Rohr JR (2018) Do host-associated gut microbiota mediate the effect of an herbicide on disease risk in frogs? J Anim Ecol 87: 489–499. 10.1111/1365-2656.12769. 29030867 PMC5812784

[ref36] Leboulenger F, Lihrmann I, Netchitailo P, Delarue C, Perroteau I, Ling N, Vaudry H (1986) *In vitro* study of frog (*Rana ridibunda* pallas) interrenal function by use of a simplified perifusion system VIII. Structure-activity relationship of synthetic ACTH fragments and γ-MSH. Gen Comp Endocrinol 61: 187–196. 10.1016/0016-6480(86)90196-6. 3007266

[ref37] Ledón-Rettig CC, Pfennig DW, Crespi EJ (2009) Stress hormones and the fitness consequences associated with the transition to a novel diet in larval amphibians. J Exp Biol 212: 3743–3750. 10.1242/jeb.034066. 19880737

[ref38] Lenth RV, Banfai B, Bolker B, Buerkner P, Giné-Vázquez I, Herve M, Jung M, Love J, Miguez F, Piaskowski J et al. (2024) *Emmeans: estimated marginal means, aka least-squares means* (version 1.10.5) [computer software]. https://cran.r-project.org/web/packages/emmeans/index.html.

[ref39] Lüdecke D, Ben-Shachar MS, Patil I, Waggoner P, Makowski D (2021) Performance: an R package for assessment, comparison and testing of statistical models. JOpen Source Softw 6: 3139. 10.21105/joss.03139.

[ref40] Luedtke JA, Chanson J, Neam K, Hobin L, Maciel AO, Catenazzi A, Borzée A, Hamidy A, Aowphol A, Jean A et al. (2023) Ongoing declines for the world’s amphibians in the face of emerging threats. Nature 622: 308–314. 10.1038/s41586-023-06578-4. 37794184 PMC10567568

[ref41] McClelland SJ, Woodley SK (2021) Water-borne corticosterone assay is a valid method in some but not all life-history stages in northern leopard frogs. Gen Comp Endocrinol 312: 113858. 10.1016/j.ygcen.2021.113858. 34302845

[ref42] McEwen BS, Wingfield JC (2003) The concept of allostasis in biology and biomedicine. Horm Behav 43: 2–15. 10.1016/S0018-506X(02)00024-7. 12614627

[ref43] McKee JK, Sciulli PW, Fooce CD, Waite TA (2004) Forecasting global biodiversity threats associated with human population growth. Biol Conserv 115: 161–164. 10.1016/S0006-3207(03)00099-5.

[ref44] Murone J, DeMarchi JA, Venesky MD (2016) Exposure to corticosterone affects host resistance, but not tolerance, to an emerging fungal pathogen. PloS One 11: e0163736. 10.1371/journal.pone.0163736. 27690360 PMC5045185

[ref45] Narayan E, Hero J-M (2011) Urinary corticosterone responses and haematological stress indicators in the endangered Fijian ground frog (Platymantis vitiana) during transportation and captivity. Aust J Zool 59: 79–85. 10.1071/ZO11030.

[ref46] Narayan EJ (2013) Non-invasive reproductive and stress endocrinology in amphibian conservation physiology. Conserv Physiol 1: cot011. 10.1093/conphys/cot011. 27293595 PMC4806611

[ref47] Narayan EJ, Forsburg ZR, Davis DR, Gabor CR (2019) Non-invasive methods for measuring and monitoring stress physiology in imperiled amphibians. Front Ecol Evol 7: 2019. 10.3389/fevo.2019.00431.

[ref48] Neely WJ, Martins RA, Mendonça da Silva CM, Ferreira da Silva T, Fleck LE, Whetstone RD, Woodhams DC, Cook WH, Prist PR, Valiati VH et al. (2023) Linking microbiome and stress hormone responses in wild tropical treefrogs across continuous and fragmented forests. Commun Biol 6: 1261. 10.1038/s42003-023-05600-9. 38087051 PMC10716138

[ref49] Pereira KE, Bletz MC, McCartney JA, Woodhams DC, Woodley SK (2023) Effects of exogenous elevation of corticosterone on immunity and the skin microbiome of eastern newts (Notophthalmus viridescens). Philosl Trans R Soc B: Biol Sci 378: 20220120. 10.1098/rstb.2022.0120. PMC10258667 37305906

[ref50] Peterson JD, Steffen JE, Reinert LK, Cobine PA, Appel A, Rollins-Smith L, Mendonça MT (2013) Host stress response is important for the pathogenesis of the deadly amphibian disease, chytridiomycosis, in Litoria caerulea. PloS One 8: e62146. 10.1371/journal.pone.0062146. 23630628 PMC3632538

[ref51] Phan TS, Schink L, Mann J, Merk VM, Zwicky P, Mundt S, Simon D, Kulms D, Abraham S, Legler DF et al. (2021) Keratinocytes control skin immune homeostasis through de novo–synthesized glucocorticoids. Sci Adv 7: eabe0337. 10.1126/sciadv.abe0337. 33514551 PMC7846173

[ref78] Piotrowski JS, Annis SL, Longcore JE (2004) Physiology of Batrachochytrium dendrobatidis, a chytrid pathogen of amphibians. Mycologia 96: 9–15. 21148822

[ref76] Raffel TR, Michel PJ, Sites EW, Rohr JR (2010) What drives chytrid infections in newt populations? Associations with substrate, temperature, and shade. Ecohealth 7: 526–536. 10.1007/s10393-010-0358-2. 21125308

[ref52] Romero LM (2004) Physiological stress in ecology: lessons from biomedical research. Trends Ecol Evol 19: 249–255. 10.1016/j.tree.2004.03.008. 16701264

[ref53] Romero LM, Wingfield JC (2015) Field techniques: measuring stress responses in wild animals. In LM Romero, JC Wingfield, eds, Tempests, Poxes, Predators, and People: Stress in Wild Animals and How They Cope. Oxford University Press, pp. 199–239.

[ref54] Sadoul B, Geffroy B (2019) Measuring cortisol, the major stress hormone in fishes. J Fish Biol 94: 540–555. 10.1111/jfb.13904. 30667059

[ref55] Santymire RM, Manjerovic MB, Sacerdote-Velat A (2018) A novel method for the measurement of glucocorticoids in dermal secretions of amphibians. Conserv Physiol 6: coy008. 10.1093/conphys/coy008. 29479435 PMC5814794

[ref56] Santymire RM, Sacerdote-Velat AB, Gygli A, Keinath DA, Poo S, Hinkson KM, McKeag EM (2021) Using dermal glucocorticoids to determine the effects of disease and environment on the critically endangered Wyoming toad. Conserv Physiol 9: coab093. 10.1093/conphys/coab093. 35186296 PMC8849142

[ref57] Scheun J, Greeff D, Medger K, Ganswindt A (2019) Validating the use of dermal secretion as a matrix for monitoring glucocorticoid concentrations in African amphibian species. Conserv Physiol 7: coz022. 10.1093/conphys/coz022. 31110770 PMC6521680

[ref58] Searle CL, Belden LK, Du P, Blaustein AR (2014) Stress and chytridiomycosis: exogenous exposure to corticosterone does not alter amphibian susceptibility to a fungal pathogen. J Exp Zool A Ecol Genet Physiol 321: 243–253. 10.1002/jez.1855. 24610865

[ref59] Selye H, Collip JB (1936) Fundamental factors in the interpretation of stimuli influencing endocrine glands. Endocrinology 20: 667–672. 10.1210/endo-20-5-667.

[ref60] Sheriff MJ, Dantzer B, Delehanty B, Palme R, Boonstra R (2011) Measuring stress in wildlife: techniques for quantifying glucocorticoids. Oecologia 166: 869–887. 10.1007/s00442-011-1943-y. 21344254

[ref61] Sjoberg DD, Baillie M, Fruechtenicht C, Haesendonckx S, Treis T (2024) *Ggsurvfit: flexible time-to-event figures* (version 1.1.0) [computer software]. https://cran.r-project.org/web/packages/ggsurvfit/index.html.

[ref62] Sjoberg DD, Whiting K, Curry M, Lavery JA, Larmarange J (2021) Reproducible summary tables with the gtsummary package. R J 13: 570–580. 10.32614/RJ-2021-053.

[ref63] Smith SM, Vale WW (2006) The role of the hypothalamic-pituitary-adrenal axis in neuroendocrine responses to stress. Dialogues Clin Neurosci 8: 383–395. 10.31887/DCNS.2006.8.4/ssmith. 17290797 PMC3181830

[ref64] Thompson LA, Spoon TR, Goertz CEC, Hobbs RC, Romano TA (2014) Blow collection as a non-invasive method for measuring cortisol in the beluga (Delphinapterus leucas). PloS One 9: e114062. 10.1371/journal.pone.0114062. 25464121 PMC4252093

[ref65] Tornabene BJ, Hossack BR, Crespi EJ, Breuner CW (2021a) Corticosterone mediates a growth-survival tradeoff for an amphibian exposed to increased salinity. J Exp Zool A Ecol Integr Physiol 335: 703–715. 10.1002/jez.2535. 34370904

[ref66] Tornabene BJ, Hossack BR, Crespi EJ, Breuner CW (2021b) Evaluating corticosterone as a biomarker for amphibians exposed to increased salinity and ambient corticosterone. Conserv Physiol 9: coab049. 10.1093/conphys/coab049. 34249364 PMC8254138

[ref67] Trumble SJ, Norman SA, Crain DD, Mansouri F, Winfield ZC, Sabin R, Potter CW, Gabriele CM, Usenko S (2018) Baleen whale cortisol levels reveal a physiological response to 20th century whaling. Nat Commun 9: 4587. 10.1038/s41467-018-07044-w. 30389921 PMC6215000

[ref68] Vukelic S, Stojadinovic O, Pastar I, Rabach M, Krzyzanowska A, Lebrun E, Davis SC, Resnik S, Brem H, Tomic-Canic M (2011) Cortisol synthesis in epidermis is induced by IL-1 and tissue injury. J Biol Chem 286: 10265–10275. 10.1074/jbc.M110.188268. 21239489 PMC3060481

[ref69] Wickham H, Averick M, Bryan J, Chang W, McGowan LD, François R, Grolemund G, Hayes A, Henry L, Hester J et al. (2019) Welcome to the Tidyverse. J Open Source Softw 4: 1686. 10.21105/joss.01686.

[ref70] Wikelski M, Cooke SJ (2006) Conservation physiology. Trends Ecol Evol 21: 38–46. 10.1016/j.tree.2005.10.018. 16701468

[ref71] Wikelski M, Romero LM, Snell HL (2001) Marine iguanas oiled in the Galápagos. Science 292: 437–438. 10.1126/science.292.5516.437c. 11330292

[ref77] Wilber MQ, Ohmer MEB, Altman KA, Brannelly LA, LaBumbard BC, Le Sage EH, Le Sage EH, McDonnell NB, Muñiz Torres AY, Nordheim CL et al. (2022) Once a reservoir, always a reservoir? Seasonality affects the pathogen maintenance potential of amphibian hosts. Ecology 20: e3759. 10.1002/ecy.3759. PMC12594070 35593515

[ref72] Wingfield JC, Romero LM (2011) Adrenocortical responses to stress and their modulation in free-living vertebrates. In Comprehensive Physiology. John Wiley & Sons, Ltd, pp. 211–234. 10.1002/cphy.cp070411.

[ref73] Woodhams DC, Brandt H, Baumgartner S, Kielgast J, Küpfer E, Tobler U, Davis LR, Schmidt BR, Bel C, Hodel S et al. (2014) Interacting symbionts and immunity in the amphibian skin mucosome predict disease risk and probiotic effectiveness. PloS One 9: e96375. 10.1371/journal.pone.0096375. 24789229 PMC4005770

[ref74] Woodhams DC, LaBumbard BC, Barnhart KL, Becker MH, Bletz MC, Escobar LA, Flechas SV, Forman ME, Iannetta AA, Joyce MD et al. (2018) Prodigiosin, violacein, and volatile organic compounds produced by widespread cutaneous bacteria of amphibians can inhibit two Batrachochytrium fungal pathogens. Microb Ecol 75: 1049–1062. 10.1007/s00248-017-1095-7. 29119317

[ref75] Wyngaard G, Chinnappa C (1982) General biology and cytology of cyclopoids. In F Harrison, R Cowden, eds, Developmental Biology of Freshwater Invertebrates. Alan R. Liss, Inc., New York, pp. 485–533.

