# Correction to: Dermal glucocorticoids are uncoupled from stress physiology and infection

**DOI:** 10.1093/conphys/coaf035

**Published:** 2025-05-10

This is a correction to: Victor Quadros, Brady Inman, Nina McDonnell, Kaitlyn Williams, L Michael Romero, Douglas C Woodhams, Dermal glucocorticoidsare uncoupled from stress physiology and infection, *Conservation Physiology*, Volume 13, Issue 1, 2025, https://doi.org/10.1093/conphys/coaf005.

In the originally published HTML version of this manuscript, the graphical abstract was missing. This error has been corrected.

