# Supplementary material for: Dermal glucocorticoids are uncoupled from stress physiology and infection
Source: Conserv Physiol. 2025 Feb 11;13(1):coaf005. doi: 10.1093/conphys/coaf005 (PMC11821355; doi:10.1093/conphys/coaf005)
Supplement: Web_Material_coaf005 [file web_material_coaf005.zip › Supplemental_Materials_Dermal_CORT_revision.pdf]

Supplemental Materials

Section 1: Newt Wash Assay and Extraction Efficiency

Section 2: Primary Glucocorticoid Identification

Section 3: Statistical Models

Section 4: Summer 2022 Eastern Newt Mesocosms Results

Section 1: Newt Wash and Extraction Efficiency

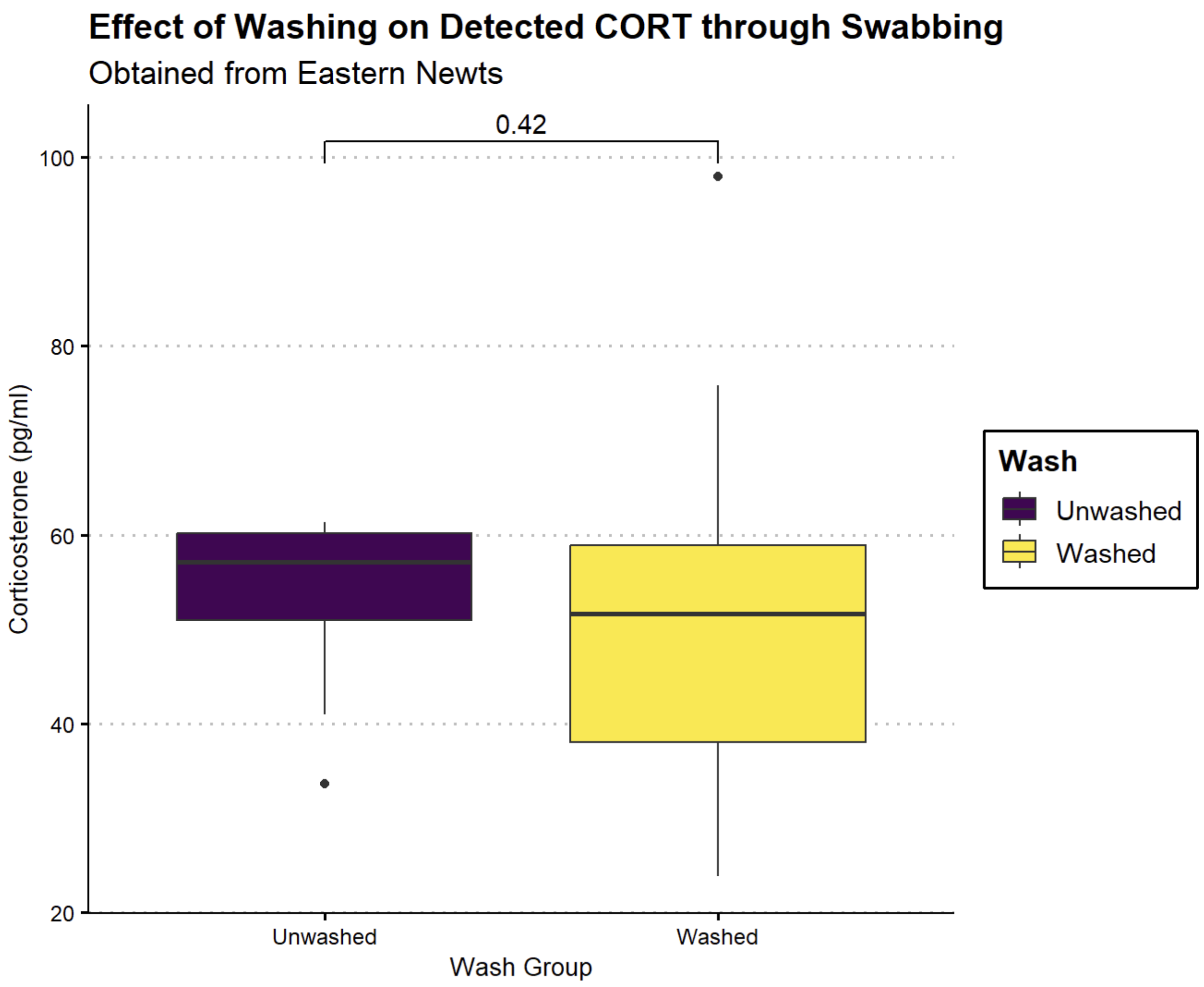

Figure S1: Boxplots showcasing the lack of an effect washing had on skin glucocorticoid swabs. The p-value is depicted between the two boxes ($p = 0.42$) and was obtained via a Welch's T-test.

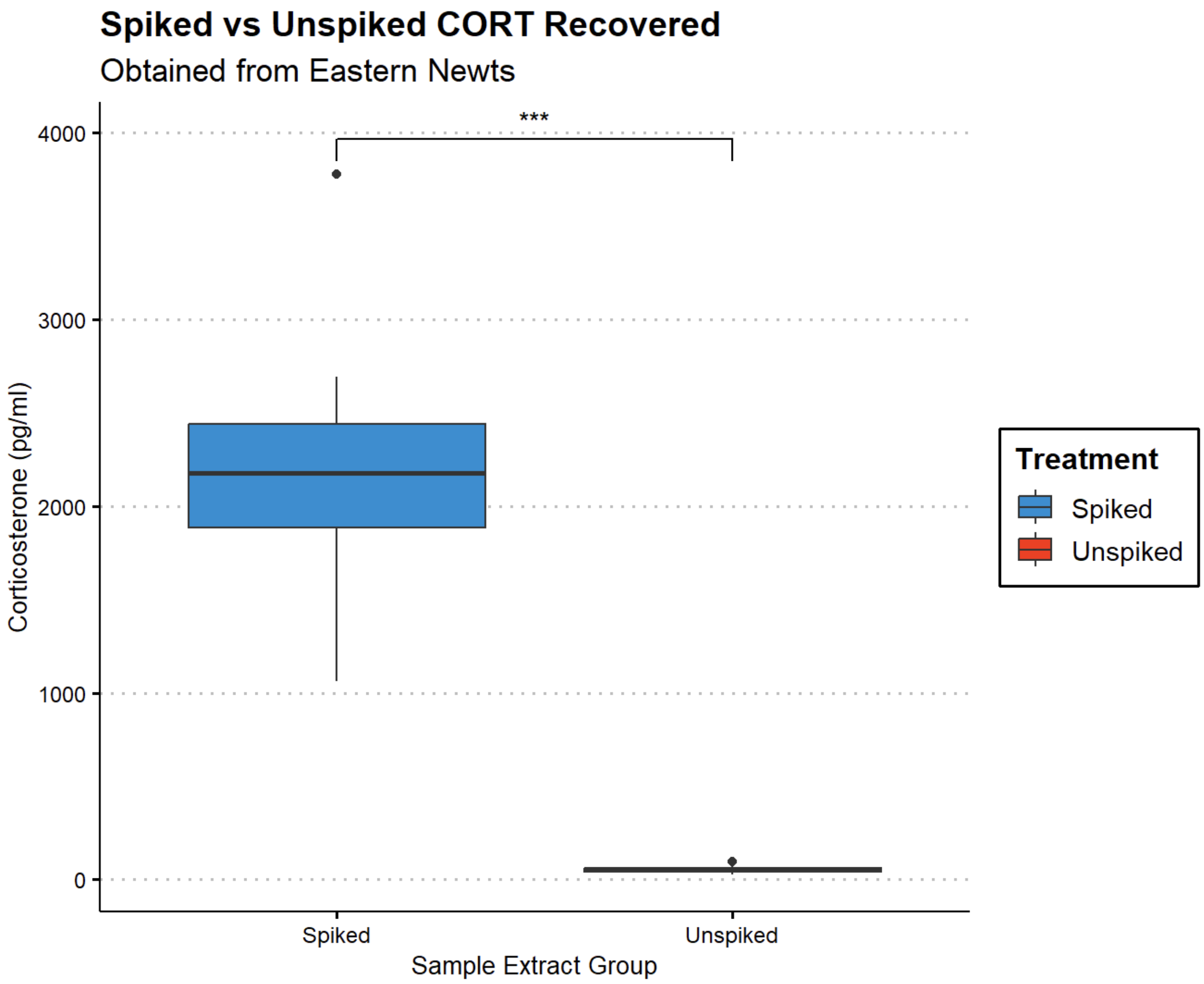

Figure S2: The following boxplots depicts the difference between swab extracts spiked with and without corticosterone (2500 pg/ml). Triple stars indicate a p-value <<< 0.05.

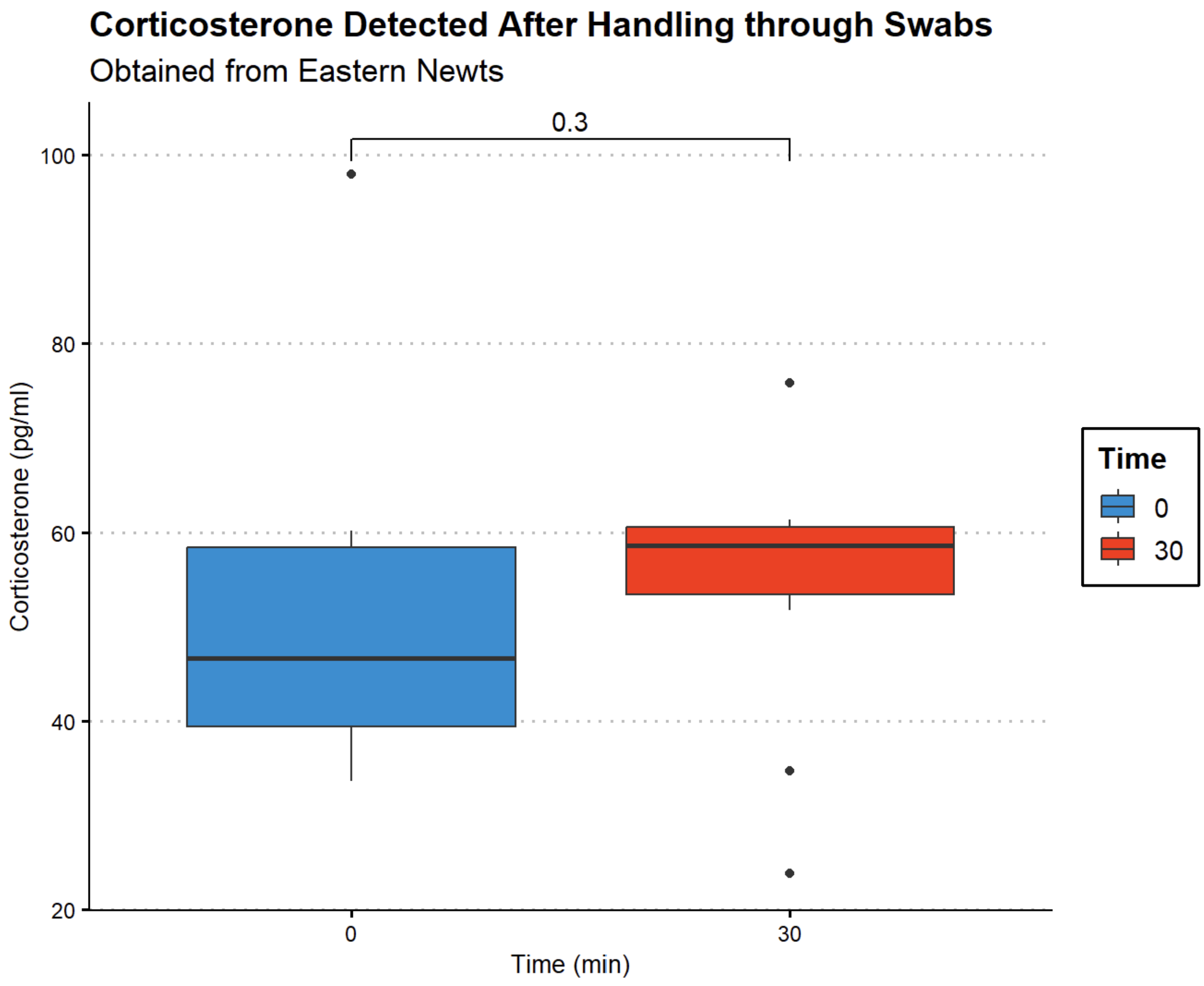

Figure S3: The above boxplots showcase the lack of a handling restraint effect on detectable corticosterone. The 0.3 between the two boxes indicates the p-value obtained via a Welch's T-test.

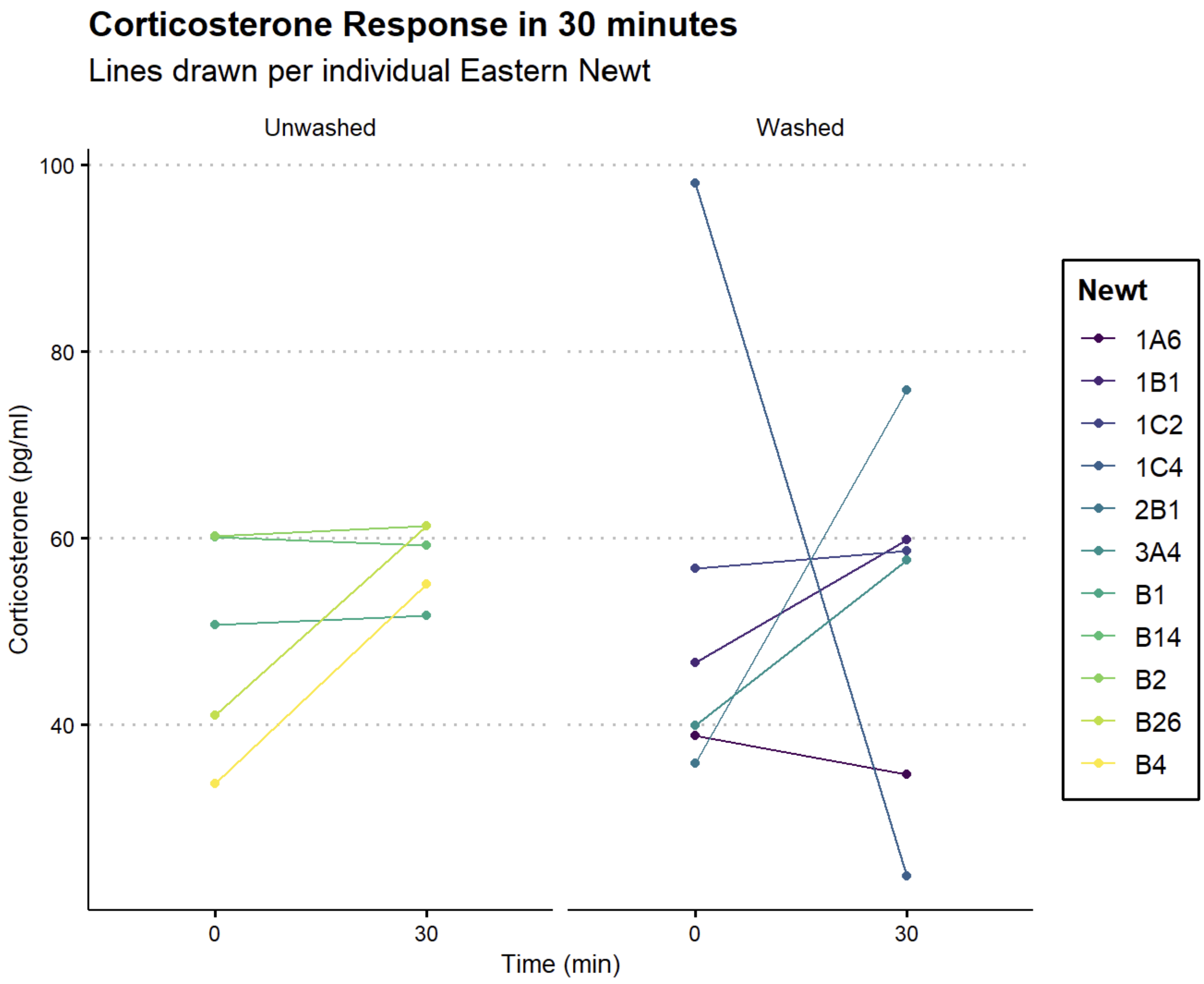

Figure S4: The following line-chart shows the change in skin corticosterone detected on individual newts. The magnitude and direction of the change in hormone is highly variable according to individual newts.

Section 2: Primary Glucocorticoid Identification

Swabs from wild leopard frogs were obtained during the fall of 2022. Frogs were swabbed in the same manner as they were during the injection challenge in 2023.

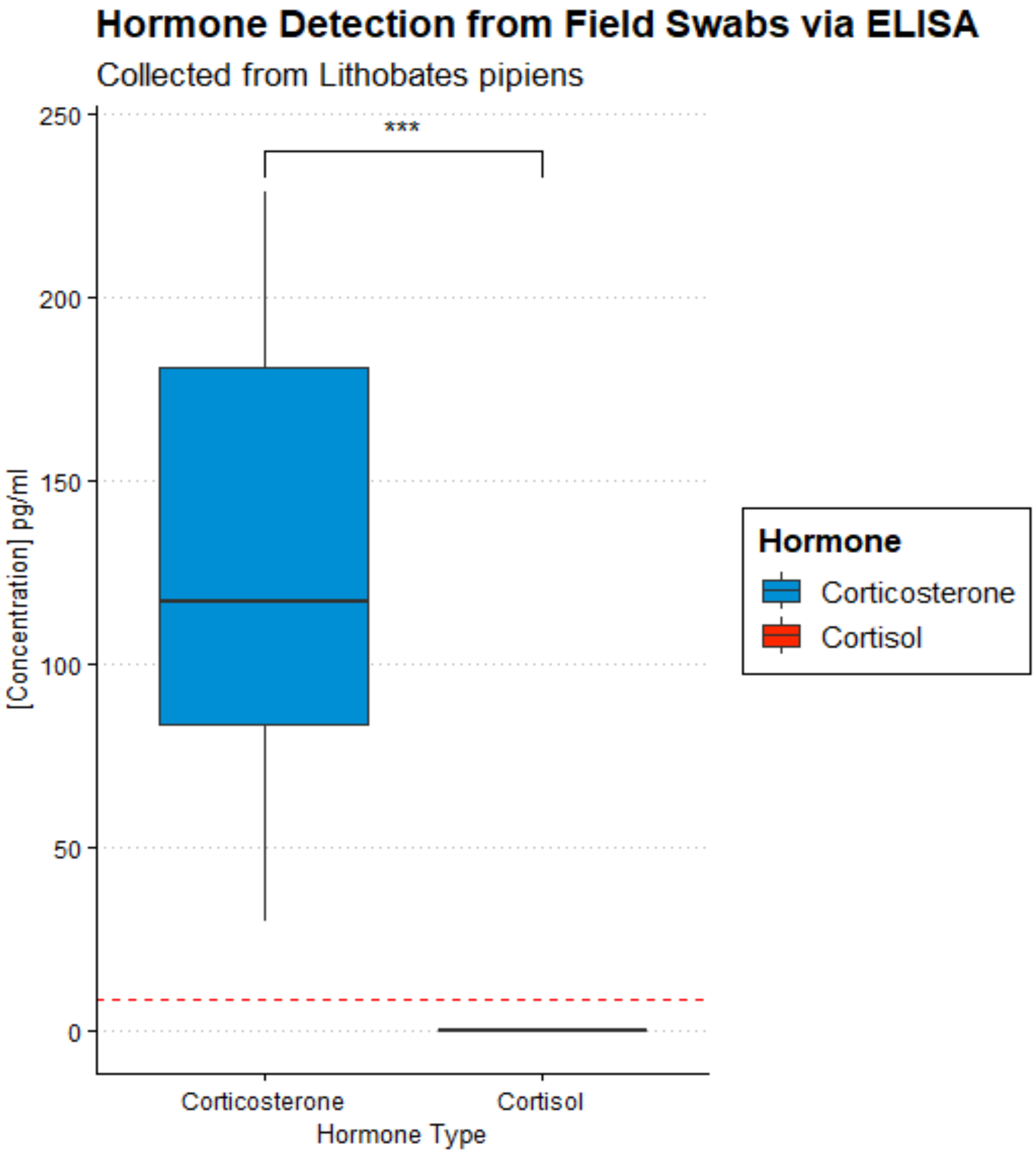

Figure S5: Comparison between corticosterone and cortisol detected from dermal swabs in the field from Northern Leopard frogs (*Lithobates pipiens*). All frogs swabbed were male (11). Triple stars between the groups represent significance levels ($p <<< 0.05$) obtained via a Welch's T-test. Dashed red line indicates lower limit of detection for the ELISA (8.8 pg/ml).

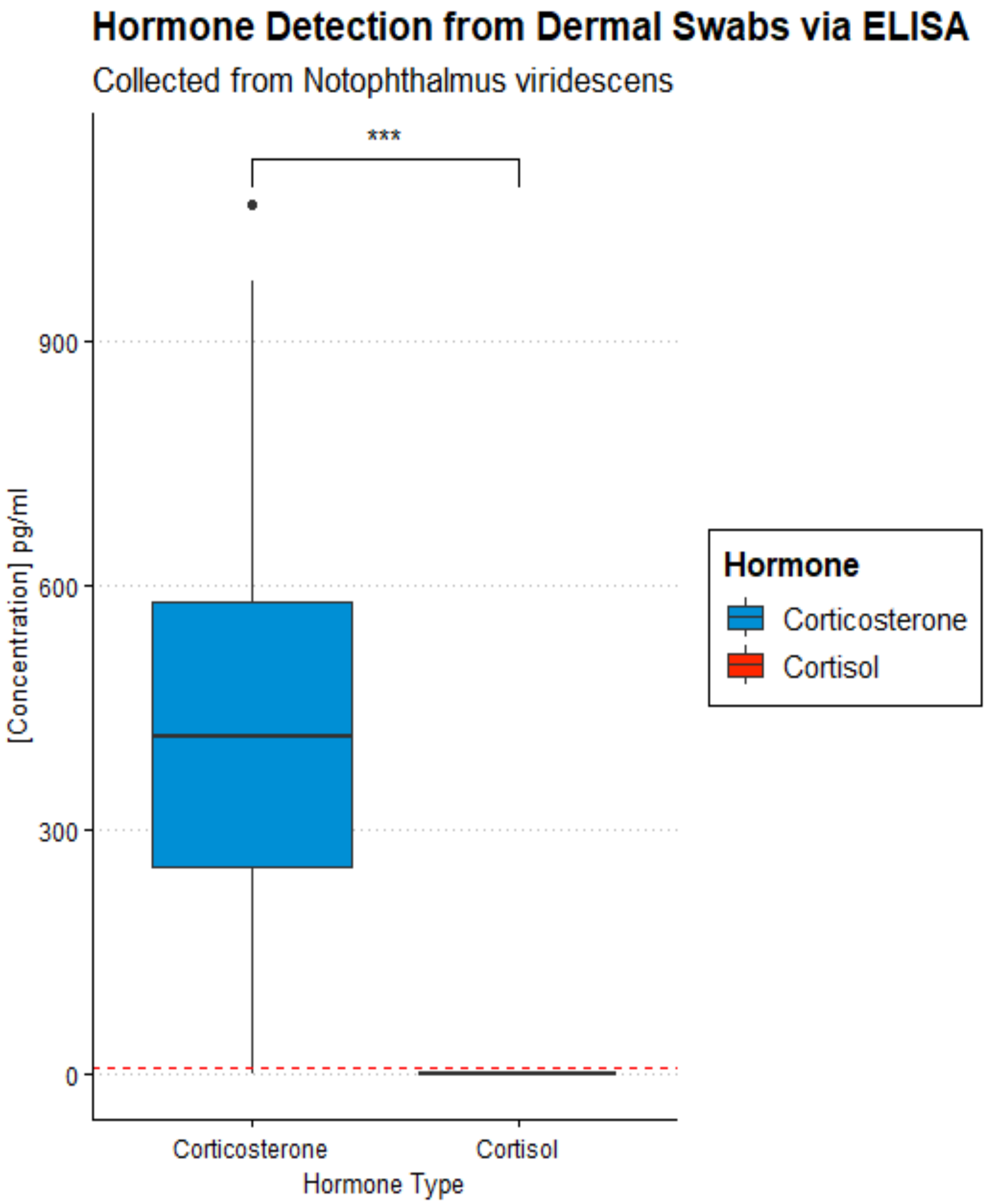

Figure S6: Comparison between corticosterone and cortisol detected from dermal swabs from 42 captive Eastern newts (*Notophalmus viridescens viridescens*). Triple stars between the groups represent significance levels (p <<< 0.05) obtained via a Welch's T-test. Dashed red line indicates lower limit of detection for the ELISA (8.8 pg/ml).

Section 3: Statistical Models

The following tables contain all of the models constructed for physiological validation and the corresponding information criteria obtained from each one. The tables are split up according to the species from which the data were collected from.

Table S1: Compiled AIC table for all models used to analyze corticosterone data obtained from eastern newts.

Eastern Newt Bd prediction models	DF	dAIC
Bd load ~ log(Corticosterone) + SVL	4	250.4
Bd load ~ log(Corticosterone)	4	267.7
Bd load ~ log(Corticosterone)*SVL	5	251.8
Bd load ~ log(Corticosterone) + SVL + (1|Newt ID)	5	2.5
Bd load ~ log(Corticosterone) + (1|Newt ID)	4	0
Eastern Newt SVL model tests	DF	dAIC
Corticosterone ~ SVL	3	0
Corticosterone ~ Weight	3	7.7
Eastern Newt Injection Models	DF	dAIC
log(Corticosterone) ~ Group + Time	10	1111.7
log(Corticosterone) ~ Group + Time + SVL	11	933.4
log(Corticosterone) ~ Group	4	1302.2
log(Corticosterone) ~ Time + SVL	9	955
log(Corticosterone) ~ Group + Time + (1|Newt_ID)	11	0
log(Corticosterone) ~ Group + Time + SVL + (1|Newt_ID)	12	4.2
log(Corticosterone) ~ Group + (1|Newt_ID)	5	200.5

Table S2: Compiled AIC table for all models used to analyze corticosterone data obtained from leopard frogs.

Leopard Frog Injection Models	DF	dAIC
log(Corticosterone) ~ Group + Time	10	2.5
log(Corticosterone) ~ Group + Time + SVL	11	3.5
log(Corticosterone) ~ Group	9	0
log(Corticosterone) ~ Time + SVL	4	60.6
log(Corticosterone) ~ Group + Time + (1|Frog_ID)	11	33.8
log(Corticosterone) ~ Group + Time + SVL + (1|Frog_ID)	12	41
log(Corticosterone) ~ Group + (1|Frog_ID)	5	73.2
Leopard Frog SVL model tests	DF	dAIC
Corticosterone ~ SVL	0.7	3
Corticosterone ~ Weight	0	3

The following outputs compile statistics obtained from the cox regression models that predicted newt survival in captivity:

Model Output 1: Survival ~ SVL

```
         coef exp(coef)  se(coef)      z Pr(>|z|)
SVL -0.009048  0.990993  0.064531 -0.14    0.888

    exp(coef) exp(-coef) lower .95 upper .95
SVL     0.991      1.009    0.8733     1.125

Concordance= 0.53  (se = 0.059 )
Likelihood ratio test= 0.02  on 1 df,   p=0.9
Wald test            = 0.02  on 1 df,   p=0.9
Score (logrank) test = 0.02  on 1 df,   p=0.9
```

Model Output 2: Survival ~ Bd load (Week 1)

```
             coef exp(coef) se(coef)     z Pr(>|z|)
`Load Acc` 0.1651    1.1795   0.1612 1.024    0.306

           exp(coef) exp(-coef) lower .95 upper .95
`Load Acc`      1.18     0.8478    0.8599     1.618

Concordance= 0.556  (se = 0.071 )
Likelihood ratio test= 1.05  on 1 df,   p=0.3
Wald test            = 1.05  on 1 df,   p=0.3
Score (logrank) test = 1.05  on 1 df,   p=0.3
```

Model Output 3: Survival ~ CORT Week 1

```
            coef exp(coef) se(coef)    z Pr(>|z|)
Cort_W1 0.004372  1.004382 0.002052 2.13   0.0331 *
---
Signif. codes:  0 '***' 0.001 '**' 0.01 '*' 0.05 '.' 0.1 ' ' 1

        exp(coef) exp(-coef) lower .95 upper .95
Cort_W1     1.004     0.9956         1     1.008

Concordance= 0.627  (se = 0.049 )
Likelihood ratio test= 3.83  on 1 df,   p=0.05
Wald test            = 4.54  on 1 df,   p=0.03
Score (logrank) test = 4.68  on 1 df,   p=0.03
```

Model Output 4: Survival ~ Mass/SVL^2

```
                coef  exp(coef)   se(coef)     z Pr(>|z|)
Body_Cond  3.086e+02 1.018e+134  1.051e+03 0.294    0.769

           exp(coef) exp(-coef) lower .95 upper .95
Body_Cond 1.018e+134 9.823e-135         0       Inf

Concordance= 0.524  (se = 0.068 )
Likelihood ratio test= 0.09  on 1 df,   p=0.8
Wald test            = 0.09  on 1 df,   p=0.8
Score (logrank) test = 0.09  on 1 df,   p=0.8
```

Model Output 5: Survival ~ Bd load in the field

```
                coef exp(coef) se(coef)     z Pr(>|z|)
`Load Field` 0.1013    1.1066   0.1002 1.011    0.312

              exp(coef) exp(-coef) lower .95 upper .95
`Load Field`      1.107     0.9036    0.9093     1.347

Concordance= 0.641  (se = 0.066 )
Likelihood ratio test= 1.02  on 1 df,   p=0.3
Wald test            = 1.02  on 1 df,   p=0.3
Score (logrank) test = 1.03  on 1 df,   p=0.3
```

Model Output 6: Survival ~ CORT in the field

```
                coef exp(coef)  se(coef)      z Pr(>|z|)
Cort_Field -0.006079  0.993939  0.005851 -1.039    0.299

            exp(coef) exp(-coef) lower .95 upper .95
Cort_Field    0.9939      1.006    0.9826     1.005

Concordance= 0.523  (se = 0.072 )
Likelihood ratio test= 1.1  on 1 df,   p=0.3
Wald test            = 1.08  on 1 df,   p=0.3
Score (logrank) test = 1.08  on 1 df,   p=0.3
```

Section 4: Summer 2022 Eastern Newt Mesocosms Results

Methods

Mesocosm Experiment

Prior to the capture of Eastern Newts, a grid of 14 mesocosms (**Image S1**)were arranged in a grid design and filled with Boston Municipal water. Water was left to stand for a week prior to the introduction of newts in addition to an inoculation of bog water obtained from the same sampling site the newts were obtained from plus leaf litter for animal welfare. Mesocosms were covered with a 40% shade cloth and were spaced roughly 5 feet apart from each other. Water temperature within the mesocosms was measured on each sampling day.

Newts were obtained from the George D. Aikens Wilderness in southern Vermont. 90 individuals were collected in total, with 42 total designated for the mesocosm experiments. Individuals were randomly placed into groups of three with a 1:2 male-to-female sex ratio in all the mesocosms. Identification was based on spot pattern, sex, and mesocosm grid number. All newts were given a three-week grace period prior to the experiment to minimize the influence of acclimation on any hormone readings. All swab samples were collected at the same time of day within three minutes of capture and identification according to the best practices for obtaining comparable glucocorticoid swabs in mammals to measure baseline hormone titers. Frozen bloodworms were fed in standardized amounts via Pasteur pipette and were always fed after experimentation on non-swab days. All animals were caught using mesh nets that were hung on the side of each mesocosm.

To manipulate baseline corticosterone levels, treatments were assigned in a Latin-squares design, wherein each row and column contained all treatment types to minimize spatial environmental

confounders. Three treatment groups were assigned to each mesocosm: a control group that received no additional acute stressor, a group that was roughly handled throughout the duration of the experiment to mimic predator interactions, and a group exposed to filtered Batrachochytrium dendrobatidis metabolites. Bd metabolites were obtained from Bd cultures grown at 20 °C in 15 ml of 1% tryptone liquid medium. Zoospore cultures at a density of 2e^6 zoospores per ml were heat killed at 60 °C for 1 hour after being reconstituted in artificial pond water. The resulting supernatant was centrifuged at 4193 (Eppendorf 5810r, 2500 rpm) for 30 minutes and filtered through a 0.22 µm syringe filter. The duration of the experiment lasted for two hours, wherein newts were caught and placed into whirlpacks filled with 100 ml of mesocosm water. Upon capture, whirlpacks for the predator and control treatment groups received an additional 100 µl of artificial pond water while the Bd metabolite group received 100 µl of the metabolite extract. Additionally, the predator group was roughly handled upon capture. All newts were left to float inside of the bags for two hours and the predator treatment group was routinely handled every 15 minutes during this period. The procedure was repeated 3 days a week for 3 weeks. On the final day of the experiment, newts were reweighed, and their snout vent lengths were measured. Snout vent lengths did not change throughout the experiment; thus, body condition was solely contingent upon changes in body mass.

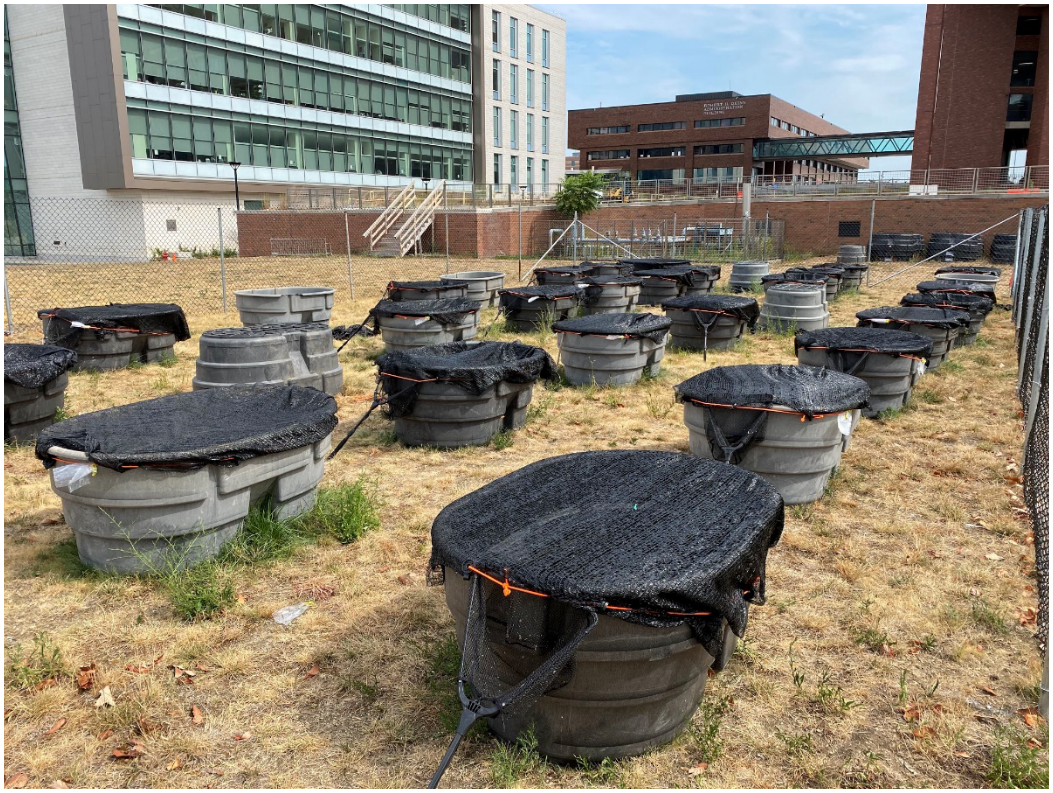

Image S1: Shown above is the mesocosm matrix used for the Summer 2022 experiment. All treatments were arranged in a Latin squares design.

Swab Collection and ELISAs

After capture, newts were handled with a new pair of gloves and dorsoventrally swabbed according to typical amphibian swabbing practices for Bd diagnosis using fine tip cotton swab. Once

collected, swab tips were placed into microcentrifuge tubes containing 500 µl of 70% ethanol solution. Swabs were vortexed and left to sit in the 4 °C fridge overnight. The following day, swab tips were removed from the ethanol solution and the microcentrifuge tubes were placed into a SpeedVac vacuum concentrator set at 60 °C for 4 hours. Once complete, the residue at the bottom of the microcentrifuge tubes was reconstituted in 200 µl of 1X PBS and stored in a -80 °C freezer. Corticosterone competitive ELISA kits were ordered from Cayman Chemical. All methods followed the protocol laid out in the manual that came with the ELISA kits; however, a serial titration of swab corticosterone extracts was performed to gauge the correct volume and concentration to utilize to fall within the linear range of the corticosterone standard. Samples were plated in triplicate and the resulting concentrations were read at a wavelength of 412 nm.Newt Wash Assay and Extraction Efficiency

Statistical Analysis

Optical densities obtained from the plate reader were copied into an excel sheet Cayman Chemical provides for calculating corticosterone concentrations. The quality of each calculated corticosterone concentration was assessed using percent coefficient of variation to determine intra-assay validity. Values below 15% were considered valid and the resulting corticosterone concentrations were collected into a single csv file for analysis using R. Considering that all individuals were sampled multiple times over the course of 4 weeks, a linear mixed model was used to determine whether both treatment groups had corticosterone values that differed from the control groups. The glmmTMB package was used to fit a multiple regression linear mixed model and confidence intervals were computed using the emmeans package. Sex, week, treatment, and newt ID were used as variables and the best fit model was determined using AIC from the AICcmodavg package. The relationship between changes in body mass and corticosterone concentrations were determined using a linear model. All model assumptions were assessed using the performance package.

Results

Over the course of the four-week experiment, 166 corticosterone swabs were sampled from all newts on each sampling day. One newt was lost half-way during the experiment due to an osprey attack while swabbing, leading to the loss of 2 potential swabs. According to the initial titration, a dilution of 1:1 of the corticosterone stock was sufficient for allowing most swabs to fall into the linear range.

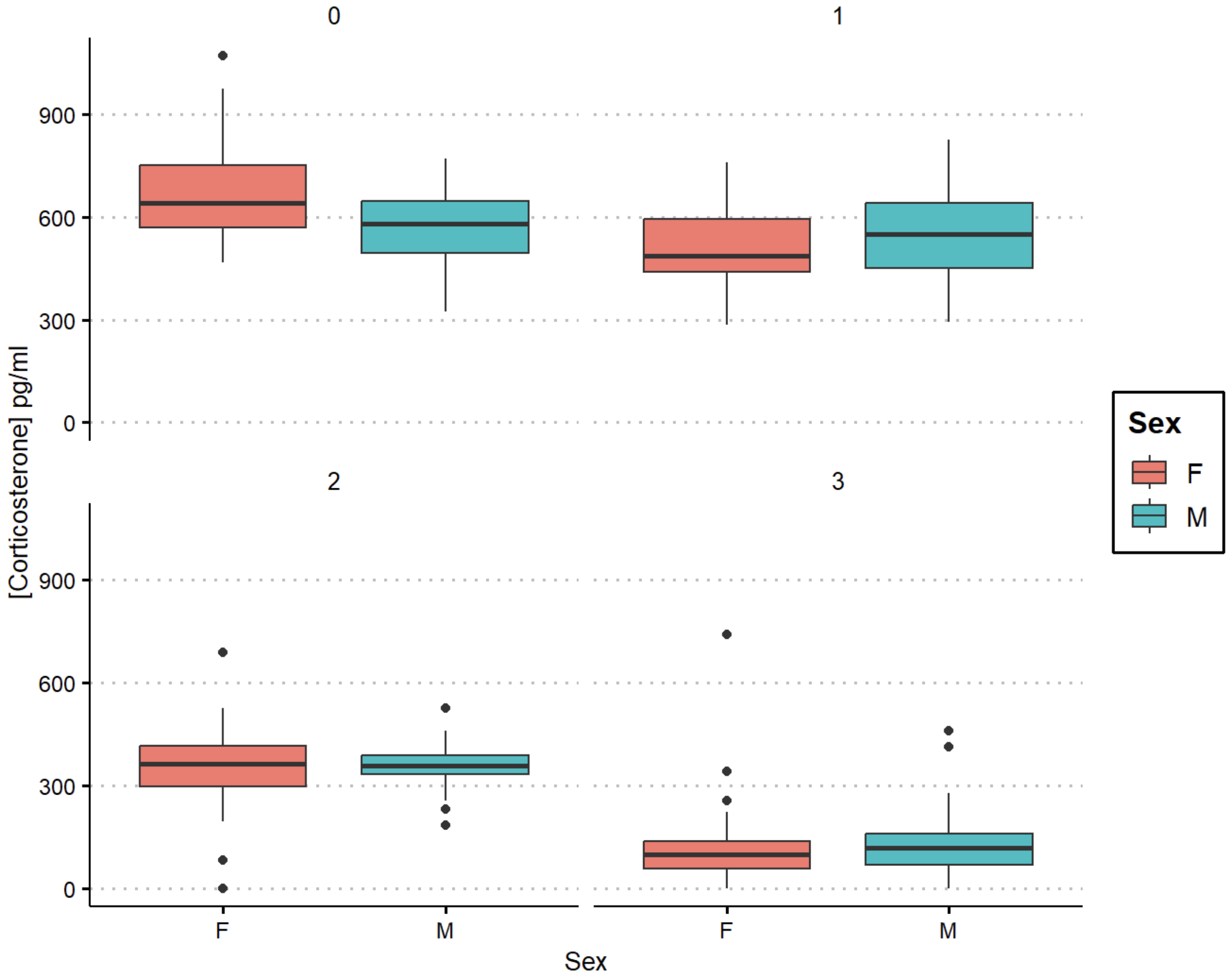

Figure S7: The effect of sex on corticosterone throughout the duration of the experiment. Overall corticosterone levels declined throughout the duration of the entire experiment, but the differences between sex were minimal or non-existent.

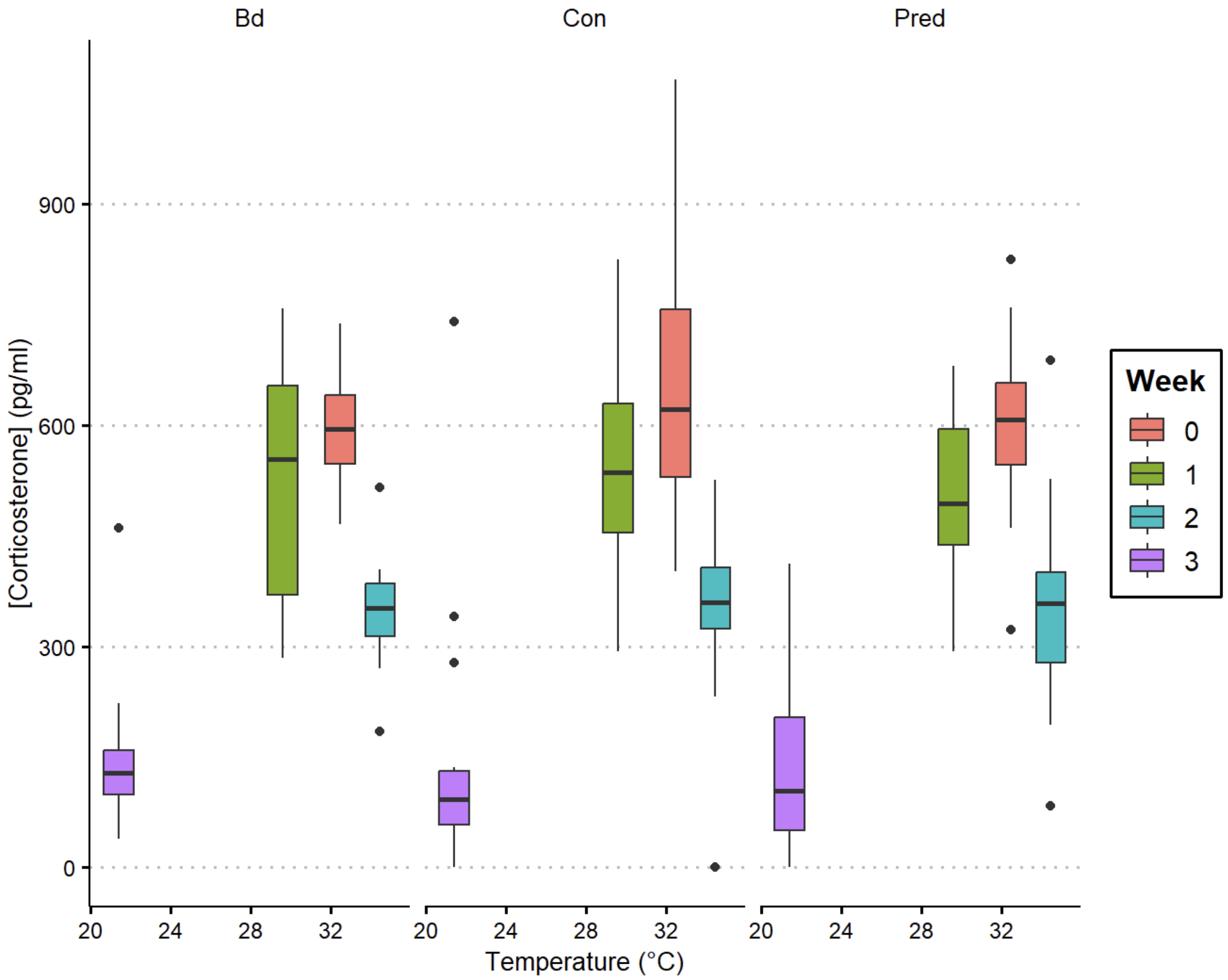

Figure S8: The impact of temperature on corticosterone across treatment groups. The same polynomial pattern can be seen across treatment groups; however, this pattern arises as a consequence of overall declining corticosterone as opposed to effects from temperature.

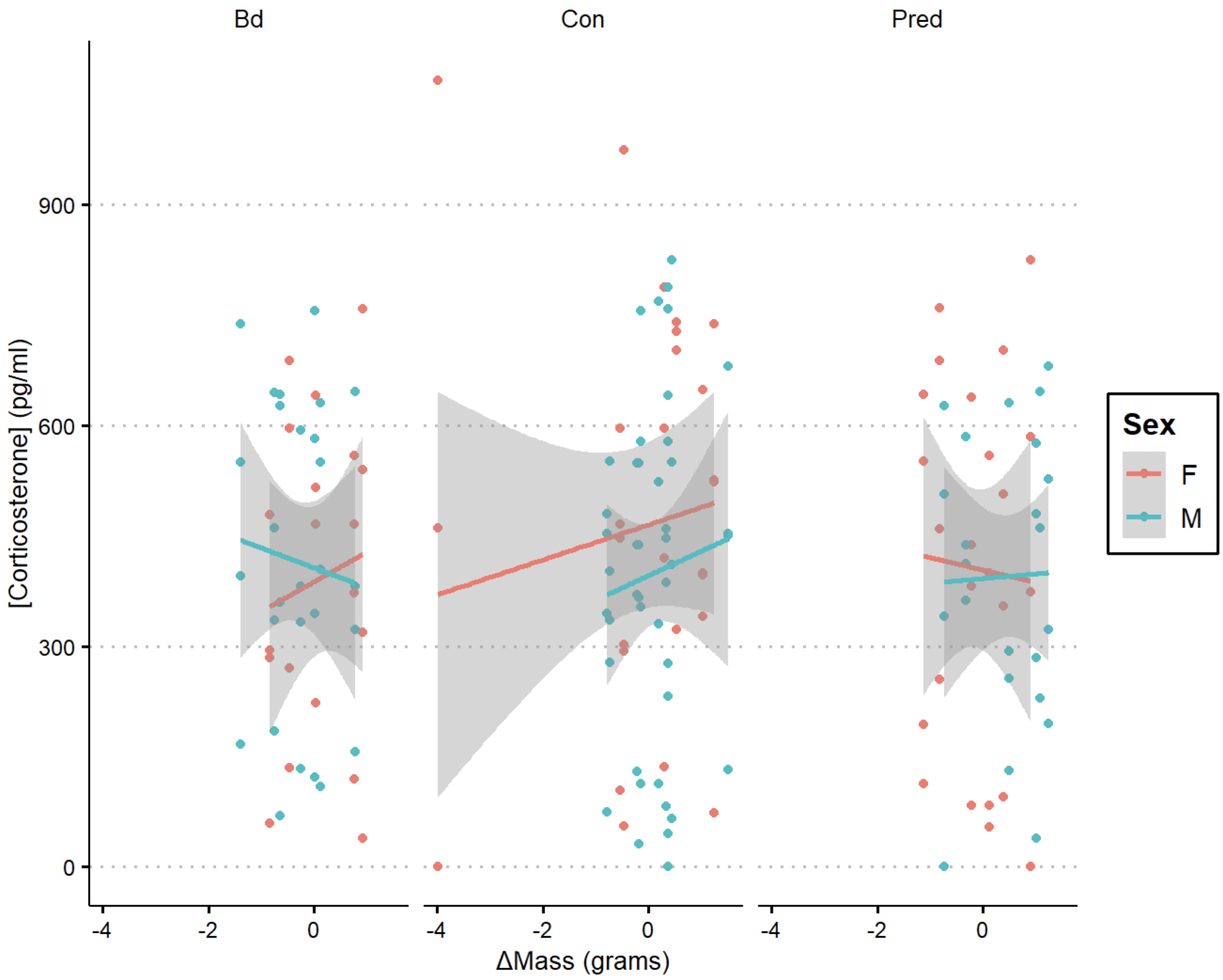

Figure S9: Linear model of corticosterone as predicted by changes in body mass across treatment groups. The slopes across all groups range from negative to positive, indicating that there is no correlation between corticosterone and body mass. Snout-vent length did not change throughout the duration of the experiment.

No differences between sexes were detected by the model. Mesocosm temperature also demonstrated no correlation with corticosterone despite massive fluctuations during multiple heatwaves. Newt mass across all mesocosms remained the same throughout the experiment based on an estimate from their initial mass before the experiment and their final masses after the experiment. Given that snout-vent length did not change, this indicates that body condition did not change. Therefore, the factors that were incorporated into the best-fit model were treatment, week, sex, and newt ID.

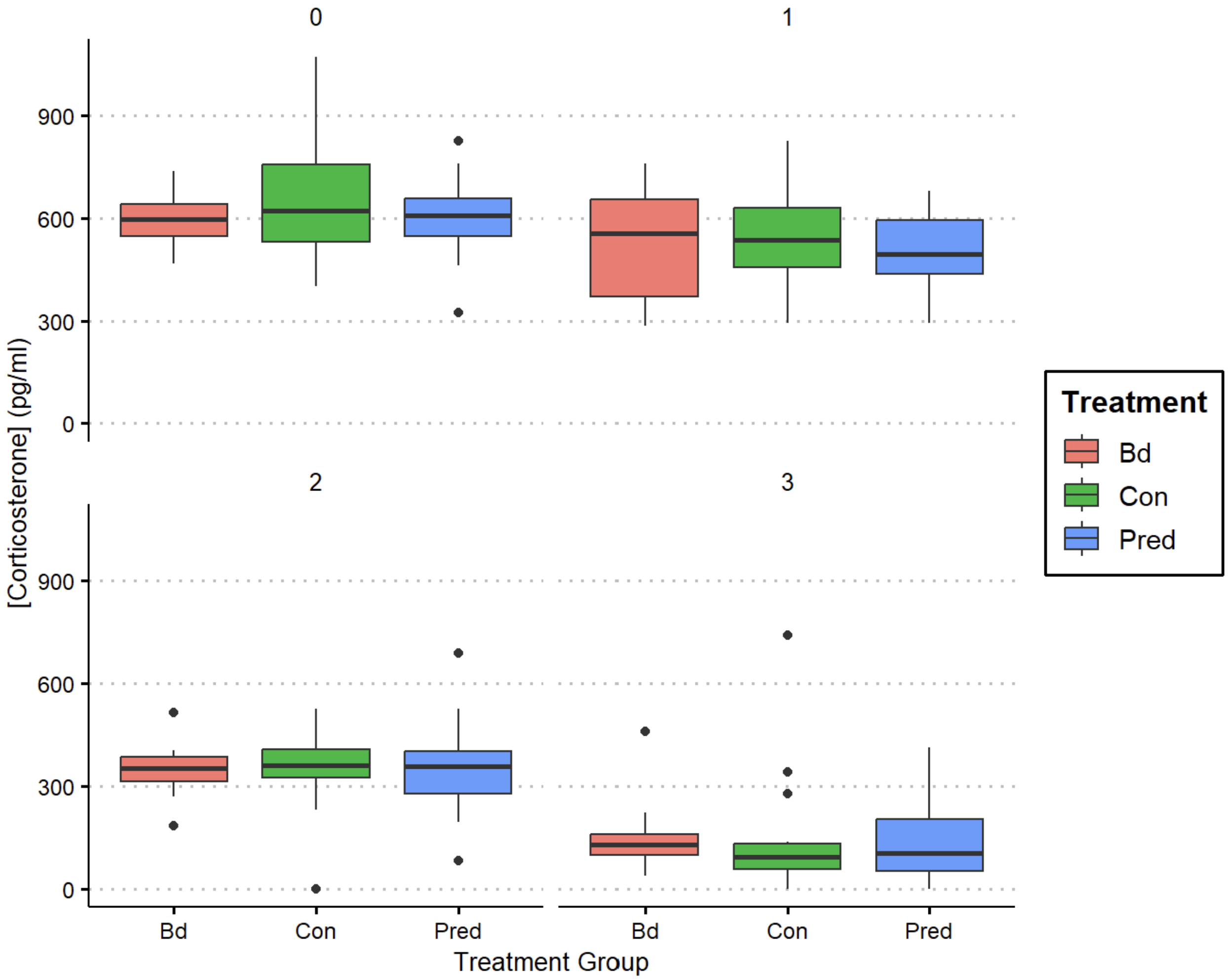

Figure S10: The effect of treatment on corticosterone through time (Weeks). The differences between each treatment group as compared to the control are minimal or non-existent. As shown in a previous figure, overall baseline levels of corticosterone fell throughout the experiment.

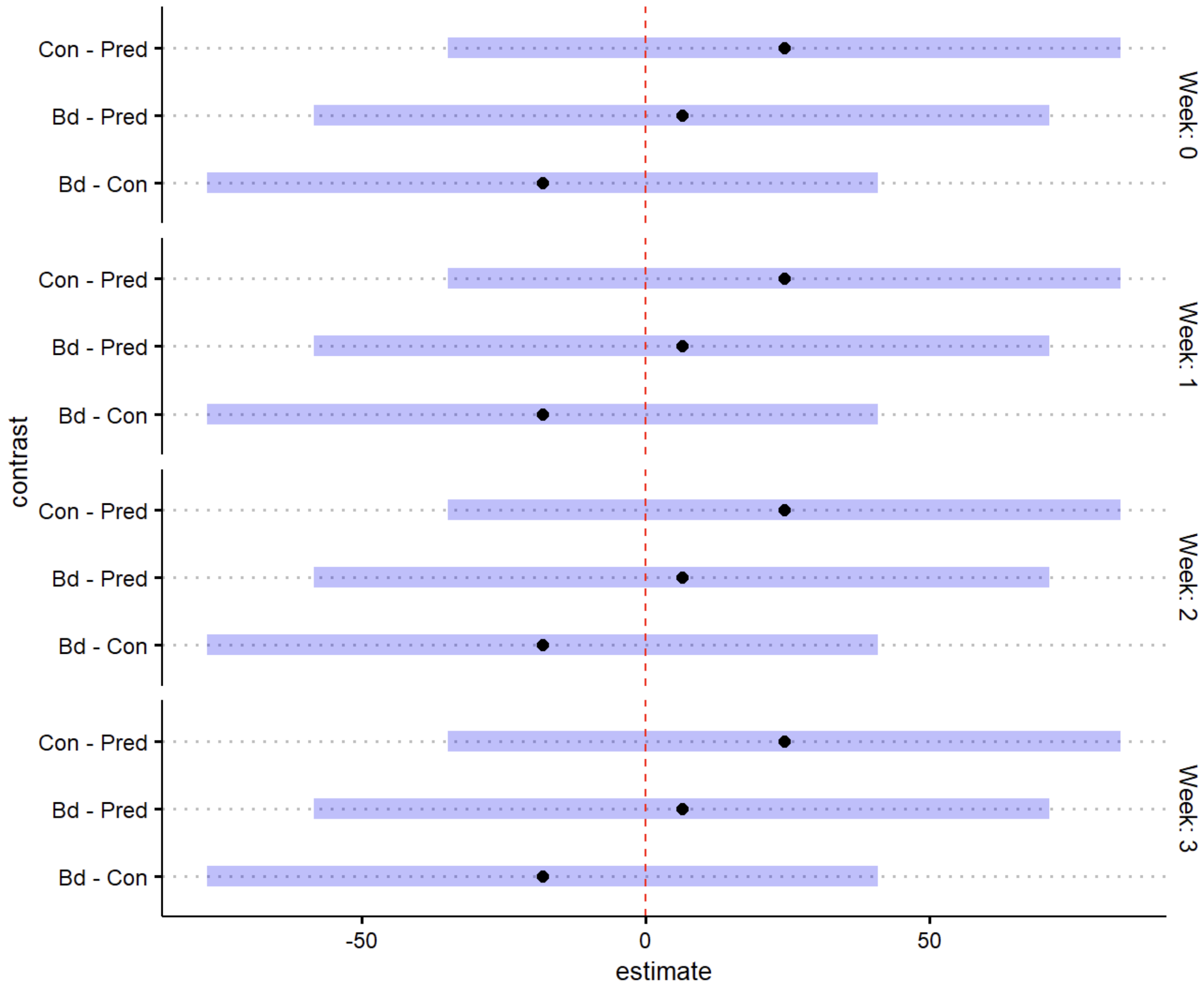

Figure S11: 95% confidence interval estimate of the mean corticosterone between each group each week as predicted by the linear mixed model. Confidence intervals that overlap with the dotted red line indicate a lack of a different between treatment groups.

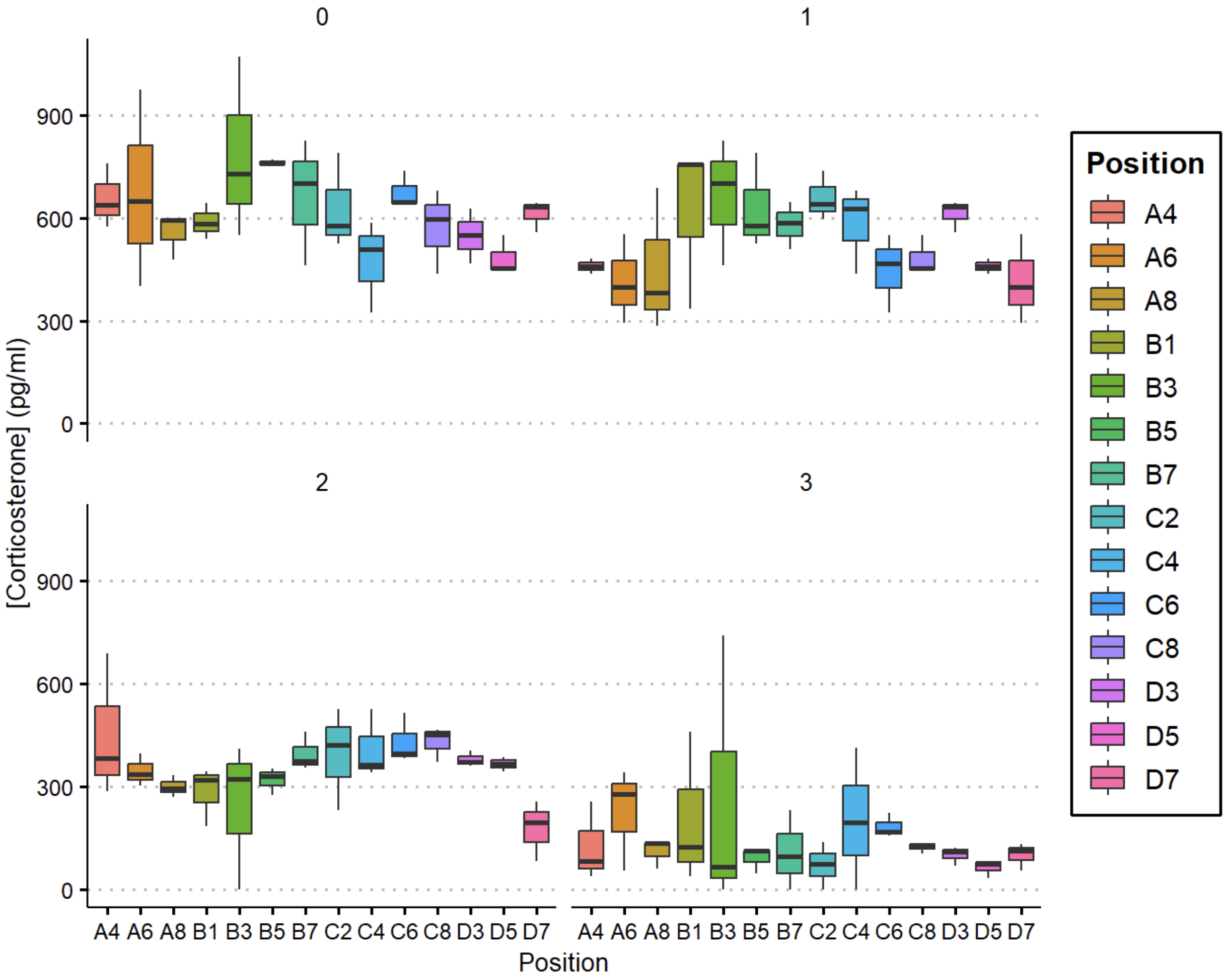

Figure S12: The influence of position within the mesocosm matrix on corticosterone across time. Once again, baseline corticosterone levels dropped throughout the entire experiment; however, there were no substantial differences between each row within the mesocosm matrix in terms of baseline corticosterone levels.

Despite experimental manipulations, no differences in baseline corticosterone were detected between treatment groups compared to the control and each other according to the model with the lowest AIC. Baseline corticosterone levels fell throughout the duration of the experiment, starting from an average of around 600 pg/ml and falling to around 150 pg/ml on the final sampling week across all treatments. Additionally, no spatial differences in baseline corticosterone within the mesocosm matrix were detected in any week throughout the experiment.
