# Supplementary figures and images for: Dermal glucocorticoids are uncoupled from stress physiology and infection
Source: Conserv Physiol. 2025 Feb 11;13(1):coaf005. doi: 10.1093/conphys/coaf005 (PMC11821355; doi:10.1093/conphys/coaf005)

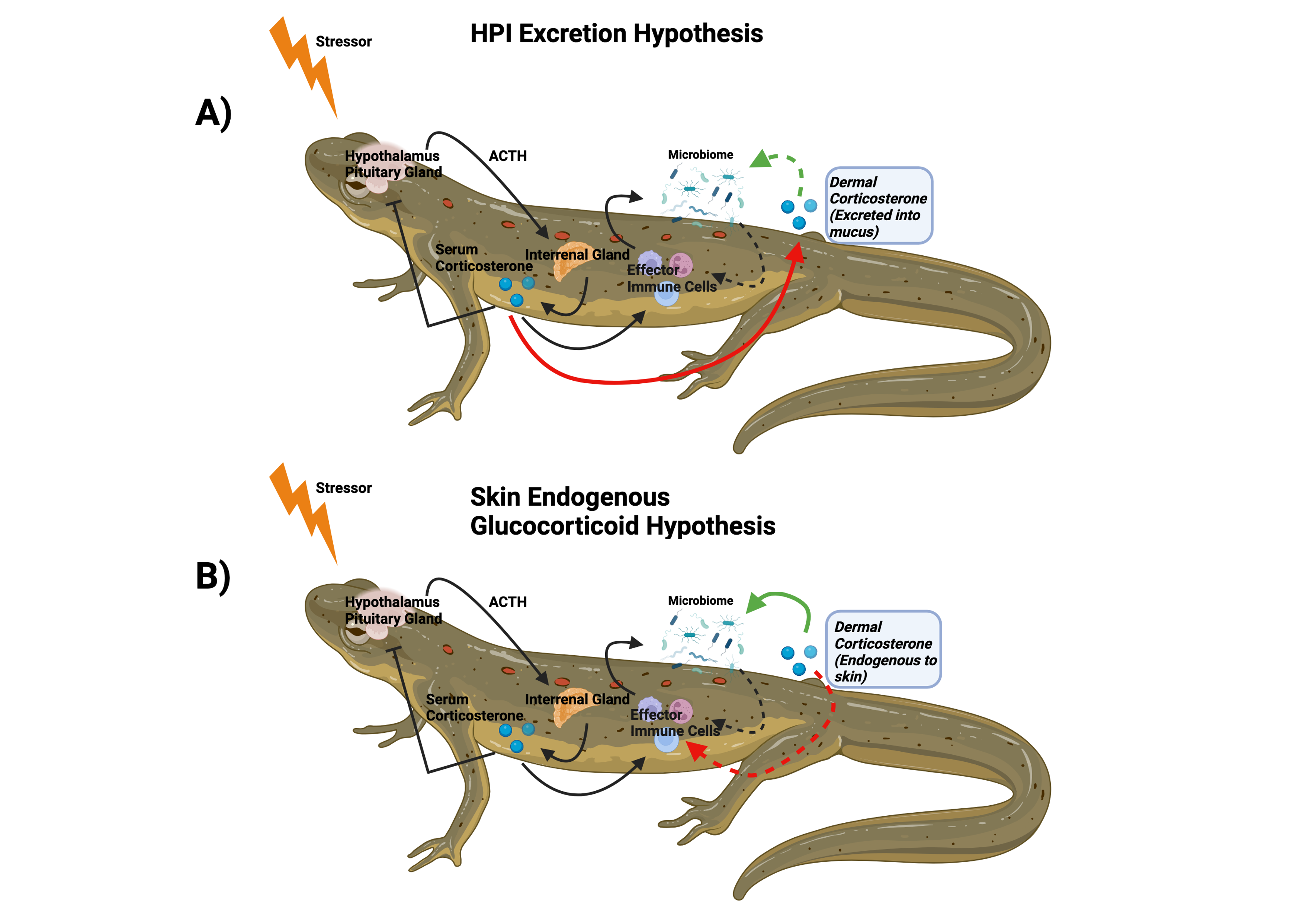

Supplement: Web_Material_coaf005 [file web_material_coaf005.zip › graphical abstract.png]
